# Modified Gold Nanoparticles to Overcome the Chemoresistance to Gemcitabine in Mutant p53 Cancer Cells

**DOI:** 10.3390/pharmaceutics13122067

**Published:** 2021-12-03

**Authors:** Eduardo García-Garrido, Marco Cordani, Álvaro Somoza

**Affiliations:** 1 Instituto Madrileño de Estudios Avanzados en Nanociencia (IMDEA Nanociencia), Faraday 9, 28049 Madrid, Spain; eduardo.garcia@imdea.org; 2 Unidad Asociada al Centro Nacional de Biotecnología (CSIC), Darwin 3, 28049 Madrid, Spain

**Keywords:** mutant p53, nanomedicine, drug delivery, cancer therapy, gold nanoparticles, nucleic acids

## Abstract

Mutant p53 proteins result from missense mutations in the TP53 gene, the most mutated in human cancer, and have been described to contribute to cancer initiation and progression. Therapeutic strategies for targeting mutant p53 proteins in cancer cells are limited and have proved unsuitable for clinical application due to problems related to drug delivery and toxicity to healthy tissues. Therefore, the discovery of efficient and safe therapeutic strategies that specifically target mutant p53 remains challenging. In this study, we generated gold nanoparticles (AuNPs) chemically modified with low molecular branched polyethylenimine (bPEI) for the efficient delivery of gapmers targeting p53 mutant protein. The AuNPs formulation consists of a combination of polymeric mixed layer of polyethylene glycol (PEG) and PEI, and layer-by-layer assembly of bPEI through a sensitive linker. These nanoparticles can bind oligonucleotides through electrostatic interactions and release them in the presence of a reducing agent as glutathione. The nanostructures generated here provide a non-toxic and powerful system for the delivery of gapmers in cancer cells, which significantly downregulated mutant p53 proteins and altered molecular markers related to cell growth and apoptosis, thus overcoming chemoresistance to gemcitabine.

## 1. Introduction

TP53 is the most frequently inactivated tumor suppressor gene in tumors, which is mutated in over 50% of human cancer types and indirectly inactivated in many others [1]. Loss of p53 tumor suppressor functions leads to the accumulation of genomic alterations culminating in cancer progression. However, in addition to the loss of the wild-type tumor suppression function, some mutant p53 (mutp53) proteins may acquire new biological properties, namely gain-of-function (GOF), which contribute to cancer progression growth, through the acquisition of oncogenic functions [2].

In many tumors, GOF p53 mutations are associated with high genomic instability, poor prognosis, reduced response to chemotherapy, promotion of migration, invasion and metastasis, and accelerated tumor recurrence [3,4,5,6,7]. Different studies have been proposed to explain the molecular mechanisms underlying the GOF proprieties of mutp53, including the modulation of the activity of several transcription factors or the inactivation of DNA damage molecular sensors [8,9]. Recently, it has been documented that DNA damage induced by gemcitabine (GEM) stabilized the nuclear localization of mutp53 proteins, which in turn triggered the expression of cell cycle-related genes, resulting in hyper-proliferation and chemoresistance [10]. In addition, mutp53 variants can alter cancer cell mitochondrial metabolism [11,12], autophagy response to various stimuli [13], and cancer microenvironment [14]. This broad spectrum of molecular properties indicates that GOF mutant p53 is involved in many different cellular pathways focused on cancer progression and aggressiveness. Hence, novel approaches aimed to inhibit the expression and function of mutp53 proteins could represent a valid therapeutic approach for cancer patients harboring mutant p53.

The pharmacological strategies currently employed for targeting mutp53 are limited to the use of small molecules (e.g., RITA, PRIMA-1, or NSC59984), which can restore the tumor suppressor function of wild-type p53 of missense-mutp53 proteins [15,16,17]. Notably, these molecules have been documented to trigger autophagy leading to mutp53 degradation and, consequently, cancer cell death [18,19,20,21].

However, despite ambitious efforts, these pharmacological treatments have been proved unsuitable for clinical application for many reasons, including problems related to delivery, drug stability, and toxicity to healthy tissues [22]. Therefore, the discovery of efficient and safe therapeutic strategies that specifically target mutp53 remains challenging.

Recently, strategies based on nucleic acids have been largely explored as a highly specific and efficacy therapy against cancer by downregulating mutp53 protein levels, which triggers apoptosis and delays cancer growth in mice [23,24]. However, although their use is promising, nucleic acids have to overcome essential obstacles limiting their therapeutic effect, including nucleic acid stability and successful delivery in vivo.

To overcome such limitations, drug delivery systems based on gold nanoparticles (AuNPs) have been successfully explored. In this sense, a variety of nanoparticles have been employed in different biomedical applications such as imaging [25], biosensing [26], and delivery of active molecules [27]. This is mainly due to their low toxicity, high biocompatibility, and easy modification, which have encouraged their use in animals [28], facilitating their translation to the clinic [29]. Moreover, the use of these nanostructures has many advantages, such as increasing the water solubility and stability of bioactive compounds and nucleic acids, leading to the increase of their blood circulation time and improving their pharmacokinetic properties [30].

In the case of oligonucleotides, the introduction of thiol moieties in their structure allows a facile functionalization of AuNPs, [31] which can be used as nanocarriers for these biomolecules. However, the selective release of this cargo in the tumour environment to produce its therapeutic effect is still a challenge to overcome [32,33,34,35]. An alternative approach is the use of positively charged polymers conjugated to the nanoparticles, which can bind the oligonucleotides through electrostatic interaction. In this regard, one of the most used polymers for this purpose is the high molecular weight branched polyethylenimine (bPEI). However, bPEI is reported to induce cytotoxicity, which severity depends on the geometry and length of the polymer, which may limit their use in cells and clinical translation [36].

In this article, new nanostructures based on AuNPs surface tailoring with modified polymers of low molecular weight bPEI (2000 MW) and a polyethyleneglycol (PEG) (3000 MW) have been developed to obtain more efficient nanocarriers. The combined use of both polymers produces highly stabilized nanoparticles with low cytotoxicity and positive potential [37]. These functionalized AuNPs, labeled as GEN1, have been used as a scaffold to produce a second generation of PEI-modified AuNPs (GEN2) through the parallel modification of bPEI with succinimidyl 3-(2-pyridyldithio) propionate (SPDP). GEN2 is a two-layer assembly consisting of two different bPEI chains crosslinked through a disulfide bond, which can be exploited as a stimulus-sensitive linker [38]. This SPDP crosslinking approach can provide similar transfection efficacy to higher molecular weight polymers [39]. Additionally, it stabilizes at the same time both the carrier and the cargo and provides a decomposition route, which can barely be achieved with other linkers [40]. As a result, GEN2-AuNPs have been used as carriers of a mixture of therapeutic nucleic acids targeting mutp53. Our system has been employed to deliver DNA-based gapmers, which present a central DNA sequence (gap) surrounded by modified RNA moiety (2′-OMe modification) [41]. Gapmers provide better inhibition activity than their unmodified analogs based on antisense oligonucleotides (ASO). In the case of gapmers, the introduction of chemical modifications at both sides of the central region improves their binding affinity, leading to stable DNA–RNA duplexes, which can be recognized by RNAse H. The recruitment of this enzyme has proven to be a successful approach to induce the cleavage of the target RNA strand [42]. Additionally, the modifications can increase their biostability, target specificity, and delivery, leading to longer effects [43].

In this regard, the gapmers herein employed have been designed to target mutated P53, which inhibition leads to a significant reduction of the expression of the mutp53 protein and cell viability. Notably, our nanoformulation reduced the chemoresistance to GEM in breast and pancreatic cancer cells carrying mutp53 proteins through the regulation of signaling pathways involved in cell growth and apoptosis.

## 2. Materials and Methods

A detailed description of the materials used can be found in the Supplementary Materials.

### 2.1. Materials

(R)-(+)-α-Lipoic acid (LP), blanched polyethylenimine (MW. 2000) (bPEI), 2,2′-dipyridyldisulfide, L-threoninol, N,N′-Dicyclohexylcarbodiimide (DCC), *N*-Hydroxysuccinimide (NHS), gold (III) chloride trihydrate, sodium citrate, citric acid, glutathione (GSH), agarose, and Hoechst 33342 were purchased from Sigma Aldrich (San Luis, MO, USA). Alpha-amino-omega-hydroxy poly(ethylene glycol) (NH2-PEG-OH MW.3091) (PEG) was provided by Iris Biotech GMBH (Marktredwitz, Germany). Dulbecco’s Modified Eagle’s Medium (DMEM), streptomycin–penicillin (100×), fetal bovine serum (FBS), l-glutamine (100×), trypsin (10×), phosphate-buffered saline (PBS), deuterated solvents, and cell culture plasticware were purchased from VWR (Radnor, PA, USA). Gemcitabine was purchased from Fluorochem (Hadfield, UK). Lipofectamine 2000 and Opti-MEM were purchased from Thermo Fisher Scientific (Waltham, MA, USA). All solvents were purchased from Scharlab (Sentmenat, Spain). Deionized water was used for polymer derivatives purification. The chemicals and the reagents have been used as received following the indications reported without further purification.

### 2.2. Experimental Procedure

#### 2.2.1. Synthesis of AuNPs

The procedure for the synthesis of gold nanoparticles of 13 d.nm size is based on Turkevitch’s method [44]. Briefly, to 100 mL of boiling water, gold (III) chloride (34 mg, 0.086 mmol) is added under vigorous stirring. Once the gold salt is dissolved and the temperature of the solution stable, a solution of sodium citrate (118 mg, 0.46 mmol) in 10 mL of water is added. The mixture is stirred at the same temperature for 15 min and then is stirred at room temperature for 12 h. Finally, the gold nanoparticles are filtrated through a 0.45 µm porous filter and stored in the fridge. Their concentration is calculated using the Beer–Lambert law from the absorbance recorded at 520 nm and the extinction coefficient of 2.7 × 10^8^ for 13 nm nanoparticles [45]. The absorbance measurement is made in triplicate and is performed using a 1:10 gold nanoparticles dilution in Milli-Q water, giving a final concentration of 8 nM. TEM images of the resulting gold nanoparticles were analysed from a measurement of 50 nanoparticles; the average diameter value obtained was 12.98 ± 1.5 nm (Figure S1). DLS measurements were analysed, obtaining an average mean hydrodynamic diameter of 19.33 ± 9.31 nm and a zeta potential of −42.3 ± 13.3 mV.

#### 2.2.2. AuNPs Multifunctionalization

Each type of AuNPs are functionalized using different ratios of the modified polymers prepared in the laboratory. The amount of each component is summarized in Table 1, and they are referred to as 1 mL of AuNPs in all cases. All the components for the functionalization are dissolved in water except 2-iminothiolane, which is dissolved in dimethylformamide (DMF).

#### 2.2.3. AuNPs GEN1

The no-functionalized AuNPs are incubated with the LP-PEG for at least 6 h at 4 °C under soft stirring before LP-PEI is added. The mixture is then left in the same previous conditions for 12–16 h. After this, the AuNPs are centrifuged at 4 °C and 13.2 K rpm. The supernatant is removed and re-dispersed with 1 mL of Milli-Q water. Repeat the process twice. After that, the resultant AuNPs (GEN1) are evaluated by DLS. DLS measurements were analyzed, obtaining an average hydrodynamic diameter of 30.79 ± 10.78 nm and a zeta potential of 12.9 ± 6.99 mV.

#### 2.2.4. AuNPs GEN2

Similar to the previous functionalization, GEN1 is incubated with Traut’s reagent (2-iminothiolane) for 6 h at 4 °C under soft stirring before PDP-PEI is added. The mixture is then left in the same previous conditions for 12–16 h. After this time, the AuNPs are centrifuged at 4 °C and 13.2 K rpm. The supernatant is removed and re-dispersed with 1 mL of Milli-Q water. Repeat the process twice. After that, the resultant AuNPs (GEN2) are evaluated by DLS. DLS measurements were analysed, obtaining an average hydrodynamic diameter of 35.02 ± 12.94 nm and a zeta potential of 35 ± 7.99 mV.

### 2.3. Oligonucleotides Experiments

#### 2.3.1. Oligonucleotides Synthesis

The oligonucleotides were prepared using a Mermade 4 DNA synthesizer and purified using Biosearch columns. The sequences of the oligonucleotides employed are described in Table 2. Nucleosides in bold contain a 2′-OMe group for increased stability and binding.

#### 2.3.2. Oligonucleotides Incubation

Any single oligonucleotide, or combination of them, was incubated following the next procedure. First, the volume of AuNPs required must be calculated based on the final molar amount of oligonucleotides and the bPEI in the nanoparticles in a relation 1:8. This volume of AuNPs is centrifuged at 4 °C and 13.2 K rpm and the supernatant is removed. The oligonucleotides are then added to the pellet and incubated for 1 h at room temperature. Then, a citrate buffer at pH 3.5 (0,1 M) is added. Its volume must be 10% of the final volume of the mixture of AuNPs and oligonucleotides. Once the buffer is added, the samples are incubated for 12 h. Then, the nanoparticles are centrifuged to remove the unbound oligonucleotides.

#### 2.3.3. Oligonucleotides Transfection

Exponentially growing cells were seeded at 5 × 10^3^ cells/well in 96-well plates or at 1 × 10^4^ cells in 12-well plates. Wild-type and mutant p53 protein expression were transiently knocked down by transfection with a mixture containing 60 pmoles of gapmers targeting different exons of the TP53 gene (p53.1-4). A scramble sequence as non-silencing control was used as a negative control. The silencing transfections were carried out for 48 h using Lipofectamine 2000 (Life Technologies), according to the manufacturer’s instructions. Cells were transfected by gapmers at a final concentration of 50 nM using Lipofectamine 2000 (Life Technologies), according to the manufacturer’s instructions. For fluorescence studies, PANC-1 cancer cells were transfected with 100 pmoles of polyT(10)FAM using Lipofectamine 2000.

### 2.4. Cell Lines and Culture Conditions

Pancreatic adenocarcinoma PANC-1 (mutant p53-R273H) and breast cancer MCF7 (WTp53) cell lines were purchased from American Type Culture Collection (ATCC, Rockville, MD, USA) and cultured in low-glucose DMEM medium with 10% FBS, 1% streptomycin–penicillin, and 1% l-glutamine at 37 °C in a Binder CB210 incubator (5% CO_2_). Triple-negative breast cancer MDA-MB-231 (mutant p53-R280K) cell line was purchased from American Type Culture Collection (ATCC, Rockville, MD, USA) and cultured in high glucose-glucose DMEM medium with 10% FBS, 1% streptomycin–penicillin, and 1% l-glutamine at 37 °C in a Binder CB210 incubator (5% CO_2_). All the procedures were performed inside a laminar flow hood Telstar CV-30/70 (Telstar, Terrassa, Spain). The list of the cell lines used in this study and their p53 status are summarized in Table 3.

### 2.5. Chemotherapy

Gemcitabine stock solution was prepared at 100 μM in DMSO. Then, different concentrations of GEM (1, 2, 4.5, 10, and 20 μM) were prepared in DMEM medium. It was incubated for 24 h with the cells, then washed 3 times with PBS and DMEM medium was added. After an additional time of 24 or 48 h, the viability assay was carried out as described in Supplementary Materials.

### 2.6. Nanoparticles Treatment

A volume of 100 μL functionalized AuNPs were added in Opti-MEM (500 μL, total volume). The cells were incubated for 24 h, washed with PBS and DMEM medium was added. After 24 or 48 h, the viability was assessed, as described in Supplementary Materials.

### 2.7. Combination Treatment

In this case, the nanoparticles were incubated, as indicated in Supplementary Materials. After 5 h, GEM (4.5 μM/well) was added and incubated for an additional 24 h. Then, the cells were washed with PBS and DMEM medium was added. The cell viability was evaluated after their incubation for an additional 24 or 48 h.

## 3. Results

### 3.1. Preparation of Multi Functionalized AuNPs

Bare AuNPs were prepared using Turkevitch’s method [44], which yielded nanoparticles with an average diameter of 12.98 ± 1.5 nm by TEM (Figure S1), a hydrodynamic diameter of 19.33 ± 9.31 nm, and a zeta potential of −42.3 ± 13.3 mV (Figure S2). Using the Beer–Lamber Law with the corresponding extinction coefficient for the diameter values obtained by TEM, it was determined to be a concentration of 8 nM. These AuNPs are coated with citrate anions that present a weak bond with the surface of the nanoparticle [46]; thus, they are susceptible to react with a variety of molecules such as sulfur or amine groups [47], although the interaction with Au-S is much stronger. For this reason, the lipoic acid (LP) was selected for the preparation of the linkers to connect the AuNPs and the polymers. This structure presents a dithiolane moiety that provides a strong binding with the gold and a carboxylic acid group that allows for an easy functionalization with the amino groups present in the selected polymers.

The polymers chosen for the functionalization are branched polyethylenimine (bPEI) of low molecular weight (MW = 2 kDa) and polyethylene glycol (PEG) (MW = 3000). This bPEI is a polymer with a large number of amino groups per monomer that can be used to increase the positive charge of the nanostructures generated, making them suitable carriers for oligonucleotides with reduced toxicity. However, the direct functionalization of the AuNPs with the modified bPEI led to an aggregation of the nanostructure, probably due to the difference of charges between the citrate-stabilized AuNP (negative charge) and the bPEI (positive charge). To overcome this limitation, PEG was introduced to stabilize AuNPs before their further modification with bPEI [48]. To ease the functionalization of the nanoparticles, both polymers, bPEI and PEG, were modified with the LP linker previously activated with NHS for a better reactivity, giving, as a result, the desired LP-bPEI and LP-PEG polymers.

**Figure 1:**
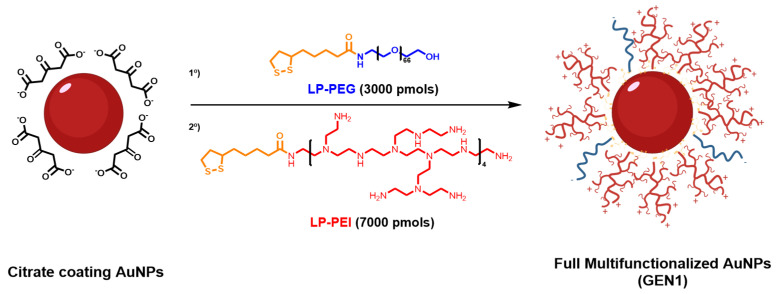
Synthesis scheme of the functionalization of citrate coating AuNPs with modified polyethyleneglycol (LP-PEG) and branched polyethylenimine (LP-bPEI).

For the functionalization of the AuNPs with the polymers, the loading of the polymers on the nanoparticles was evaluated. For this purpose, different amounts of LP-PEG, the stabilizing agent, were added and the zeta potential assessed. It was observed that for 1 mL of AuNPs, the hydrodynamic size increased from to 30.79 ± 10.78 nm (Figure S3B), and the z-potential became gradually more positive until it reached 10,000 pmols of LP-PEG. After this point, the zeta potential did not change, and it was considered the saturation point of the AuNPs (Figure S3A). On the other hand, the minimum amount of LP-PEG required to stabilize the AuNPs was 3000 pmols. Thus, the optimum formulation will imply the use of 3000 pmols of LP-PEG and 7000 pmols of LP-bPEI (Figure 1). This first generation of functionalized AuNPs with these amounts of polymers (GEN1) was centrifuged to remove the unreactive polymers and resuspended in 1 mL of water to be characterized by DLS. In comparison with the non-functionalized AuNPs, GEN1 shows a great change in the zeta potential from to 12.9 ± 6.99 mV (Figure S2B). However, the hydrodynamic size does not present remarkable changes with respect to the AuNPs functionalized only with LP-PEG. These results suggest that the polymer LP-PEI has been incorporated and has a profound effect on the z-potential. The stability of GEN1 was evaluated through the z-potential and hydrodynamic size values obtained at different periods of time at room temperature and 4 °C, being stable after 3 weeks and 6 months, respectively.

A second generation (GEN2) of PEI-based AuNPs was prepared, based in GEN1. It required the addition of a second molecule of bPEI to GEN1. The GEN2 was expected to provide a higher positive z-potential, which should increase the overall stability and the oligonucleotide binding, without an increase in toxicity (Figure S4) as is expected for larger bPEIs. To prepare this derivative, SPDP was employed to modify the amines of bPEI. SPDP is a widely used heterobifunctional crosslinker due to the presence of an NHS-ester and a pyridyldithiol group that allows a good reactivity with amines and sulfhydryl elements. On the other hand, thiol moieties were introduced in the amine groups of GEN1 nanoparticles. Then, the combination of these modified units yielded the desired GEN2, in which bPEI molecules were connected through a disulfide moiety. This type of bond is robust but can be easily broken in the presence of glutathione, which is present inside cells at high concentrations, particularly in tumoral cells [49]. Thus, the oligonucleotides bound to the nanoparticle could be released more efficiently inside tumoral cells.

**Figure 2:**
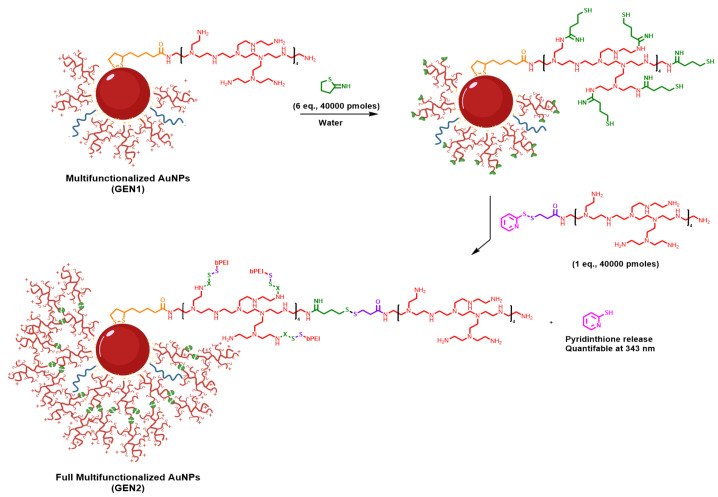
Synthesis scheme of GEN1 AuNPs activation of GEN1 2-iminothiolane to consecutively being functionalized with SPDP-bPEI to obtain GEN2 AuNPs.

First, bPEI was modified with the SPDP crosslinker to obtain SPDP-bPEI as functionalizing agent (Figure 2). AuNPs GEN1 were incubated with 2-iminothiolane to introduce thiol groups, and SPDP-bPEI was added to complete the functionalization. After this, the nanoparticles were centrifuged and the supernatant was removed and analyzed by UV to quantify the pyridine-2-thione (λ = 343 nm, ε = 8080 cm^−1^) released during the process (Figure S5). This data was used to determine the yield of functionalization, 35%, which suggests steric hindrance due to the proximity between polymers. The pellet was resuspended with water to the original volume, and the new GEN2 AuNPs were characterized by DLS.

GEN2 AuNPs presented a similar size by DLS as GEN1, 35.02 ± 12.94 nm (Figure S3C) but a significant increment in the zeta potential from to 35 ± 7.99 mV. This strategy was repeated over the GEN2 to obtain a new generation of these AuNPs (GEN3), but unfortunately, the yield of the process was lower than 10%, with a slight zeta potential increment (Figure S6) and a significant destabilization of the nanoparticles. Particularly, the GEN3 was stable at room temperature for 4 days and 2 weeks at 4 °C. For these reasons, GEN3 was not further assessed, and the main studies were carried out with GEN2.

In this regard, the size and z-potential of GEN2 in the presence of different concentrations of glutathione (GSH) were evaluated. Thus, GEN2 was incubated in the presence of GSH at different concentrations, 1 µM and 1 mM, emulating an extra and intracellular environment (Figure S7A,B). No significant changes in the first 2 h were observed, but after this time, some changes in size were appreciated and, more notably, in the potential of GEN2 at 1 mM. It was finally observed that while the ones at 1 µM did not experience important changes, the one incubated at 1 mM reverted to a stage very similar to GEN1, which indicates a good release of the SPDP-bPEI. Due to all these factors, GEN2 was chosen to test the oligonucleotide binding.

### 3.2. Transfection Efficacy of GEN2-AuNPs

Before its use in cell culture, the binding to oligonucleotides of GEN2 was tested. The oligonucleotide (PolyT(10)FAM) used in this study consists of a single chain of ten units of thymine with a fluorescein (FAM), a green fluorescent molecule, at the 5′-. This combination was chosen due to the low affinity of the T nucleobase to the gold surface [50], avoiding indirect electrostatic binding, and the ease of tracking the molecule due to the presence of FAM [51].

Thus, the PolyT(10)FAM was incubated with GEN2 AuNPs. After centrifugation, the resulting pellet and supernatant were analyzed by a gel retardation assay to quantify the amount of oligonucleotide in each one. During the incubation time, a citrate buffer was added to lower the pH of the solution to increase the binding between the amines of the GEN2 and the phosphates of the oligonucleotide [52]. This incubation method was evaluated under different factors, such as bPEI/oligonucleotide molar ratio, pH, and buffer concentration. Regarding the last two factors, the best results were obtained at 3.5 pH and buffer concentration of 10 mM, as is observed in the analysis of the agarose gels of GEN2 AuNPs (Figure S8A–C). This approach is based on the low pH assisted method, commonly employed to reduce the repulsion between oligonucleotides nucleobases and AuNPs [53,54]. These factors contribute to the molecules of PEI reaching their maximum protonation state, and therefore, enhance their electrostatic binding capacity [55,56]. Fixing these values achieved the conjugation of the 67% of the oligonucleotide at molar ratios PEI/oligonucleotides of at least 8. This means an N/P ratio of 34 (calculated as N/P ratio = 7.53 × weight ratio of PEI/DNA [57]), similar to the optimal N/P ratio recently calculated for PEI 25,000 MW [58,59]. These values represent a significant improvement if the difference in the molecular weight between them is considered. Thus, GEN2 AuNPs at those conditions were chosen to test their biological applications as carriers.

### 3.3. Biological Activity of GEN2-AuNPs

Once the AuNPs were synthesized, we assessed their biological activity in different cancer cell lines carrying mutant or wild-type p53.

**Figure 3:**
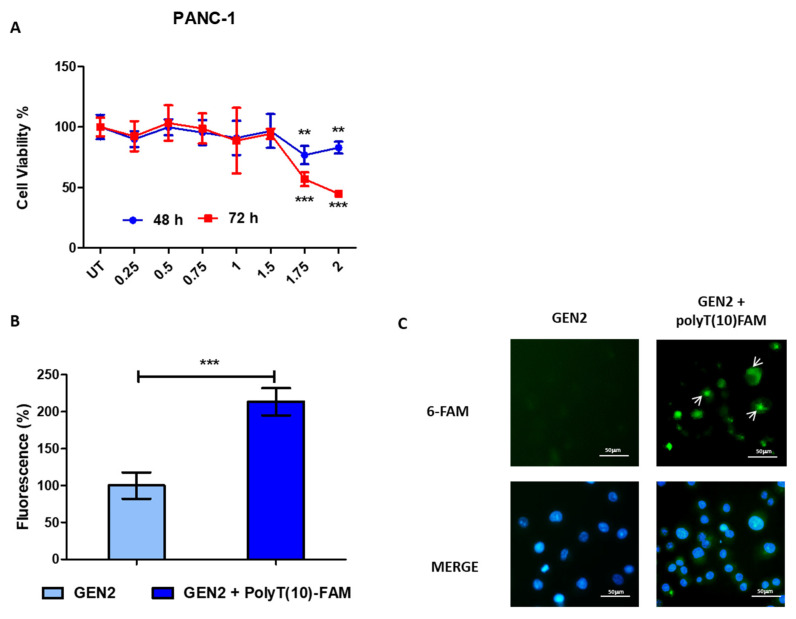
Biological activity of GEN2-AuNPs. (**A**). PANC-1 pancreas cancer cell lines were seeded in 96-well plates, incubated with GEN2-AuNPs for 48 and 72 h at the concentrations indicated, ranging from 0.25 to 2 nM, and the viability was assessed. The values of treated cells were normalized with untreated controls and reported as mean ± SE. (**B**) The PANC-1 pancreas cancer cells were incubated with GEN2 -AuNPs-polyT(10)FAM for 24 h. The fluorescence was measured with a multi-well plate reader and normalized to that of control (GEN2) and reported as mean ± SD. (**C**) Representative fluorescence images of PANC-1 cancer cells treated with GEN2 and treated with GEN2-polyT(10)FAM. FAM in green and nucleus are labeled in blue by Hoechst staining. Statistical analysis was performed using one-way ANOVA (each group vs. control). (*** *p* < 0.001, ** *p* < 0.01).

Increasing concentrations of AuNPs, ranging from 0.25 nM to 2 nM, were incubated with PANC-1 pancreas cancer cells (Figure 3A) and the breast cancer cell lines MDA-MB-231 and MCF-7 (Figure S9A,B), and cell viability was assessed using the alamarBlue assay after 48 h and 72 h.

For PANC-1 and MCF-7 cell lines, the reduction of cell viability was negligible until the concentration of 1.75 nM, whereas in MDA-MB-231 cancer cells, the viability was affected at a concentration of 1.5 nM. Later, we incubated PANC-1 and MCF-7 cancer cells with GEN2 AuNPs-polyT(10)FAM, and after 24 h, we measured the 6-FAM fluorescent intensity using a multi-well plate reader to evaluate the internalization of the oligonucleotide meditated by the nanoparticle (Figure 3B and Figure S10A). Interestingly, we observed a significant increase in 6-FAM fluorescence in both cell lines tested, suggesting that after intracellular stimulus, our modified AuNPs were able to release polyT(10)FAM in cellular space. The 6-FAM activity was confirmed by fluorescence microscopy (Figure 3C and Figure S10B).

As a control condition, polyT(10)FAM was transfected both individually and complexed with liposomes in PANC-1 and MCF-7 cancer cell lines, and the fluorescence intensity was assessed after 24 h (Figures S11 and S12). As expected, liposomal transfection strongly increased 6-FAM fluorescence in such cell lines, as confirmed by multi-well plate reader and fluorescence microscopy.

### 3.4. Mutant and Wild Type p53 Cancer Cells Show Different Sensitivity to Chemotherapy

To evaluate the different responses of cancer cell lines to GEM, we assessed the cell viability in mutant and wild-type p53 cancer cells after incubation with increasing concentrations of GEM for 48 and 72 h. In accordance with previously published data [10,13], PANC-1 and MDA-MB-231 cancer cells bearing mutant p53 proteins showed less sensitivity to GEM treatment with respect to MCF-7 cancer cells and their viability was reduced between 15–30% after 72 h of incubation (Figure S13A,B). In contrast, GEM drastically affected the viability of MCF-7 cancer cells, which showed a survival rate of 40% after 72 h from the treatment (Figure S13C).

To better understand the mechanism behind the chemoresistance to GEM in mutant p53 cancer cells, some molecular markers related to apoptosis and cell growth in PANC-1, MDA-MB-231, and MCF-7 cancer cells were evaluated by Western blots (Figures S13D and S14). Firstly, we assessed the effect of the GEM on p53 protein levels. We observed a significant increase in p53 protein levels in both mutant and wild-type p53 cancer cell lines following the treatment with GEM (4.5 µM) for 72 h. This observation is congruent with previous studies describing the interplay between DNA damage and p53 activity [60,61,62] and suggests the existence of a mechanism leading to the stabilization and induction of p53 in response to DNA damaging agents. Later, the level of the antiapoptotic protein Bcl-2 was assessed. This protein contributes to cancer formation and progression by promoting the survival of cancer cells and represents a canonical target for cancer therapy [63]. Interestingly, an increase of Bcl-2 was observed when PANC-1 cells were treated with GEM, whereas no significant reductions in this protein were observed in MDA-MB-231 and MCF-7 breast cancer cells.

Then, we evaluated the activation of mTOR signaling after GEM treatment. It is worth mentioning that hyperactivation of mTOR signaling represents one of the mechanisms responsible for chemoresistance in cancer, and its inhibition is exploited by chemotherapy drugs to exert their anti-tumor action [64]. We observed that in PANC-1 cancer cells, GEM induced the phosphorylation at Ser371 of p70S6 protein (Figures S1 and S14), indicating the activation of the mTOR pathway and chemoresistance, whereas, in MDA-MB-231 cells, its modulation was negligible. Interestingly, in MCF-7, a substantial reduction in the mTOR pathway was observed, indicating their sensitivity to chemotherapy treatment.

### 3.5. Modified Gold Nanoparticles Reduce Mutant p53 Cancer Cell Proliferation

Several studies have attributed to GOF of mutant p53 proteins its involvement in the repression of autophagic cell death [13] and in the stimulation of cell growth [9]. This occurs through the upregulation of several cyclins and cdk1-associated kinases activities, which lead to a mutant p53/NF-Y-dependent increase in DNA synthesis [9].

**Figure 4:**
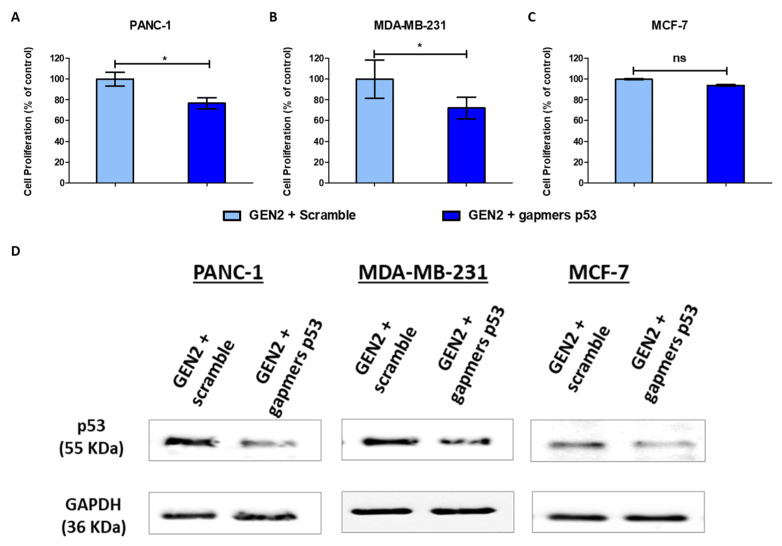
Modified gold nanoparticles reduce mutant p53 cancer cell proliferation. (**A**–**C**) The cells lines were seeded in 96-well plates, incubated overnight, and treated with GEN2-AuNPs functionalized with gapmers against p53. After 72 h, their viability was assessed. (**D**) Whole-cell extracts were processed for Western blot analysis of the indicated antibodies. GAPDH protein level in the same extract was used as a control loading. Statistical analysis was performed using one-way ANOVA (each group vs. control, * *p* < 0.05).

To assess the therapeutic potential of GEN2-AuNPs, we functionalized them with a mix containing four gapmers targeting the TP53 gene (Table 2). Then, these nanocomplexes were incubated for 72 h with cancer cell lines carrying mutant and wild-type p53 proteins, and the cell viability was measured using the alamarBlue assay. Interestingly, the treatment with AuNPs was able to effectively reduce cell viability in PANC-1 and MDA-MB-231 mutant p53 cells by approximately 25%, whereas it did not show a significant effect on MCF-7 cancer cells (Figure 4A–C). We also confirmed by Western blot assay a reduction of the levels of p53 proteins (both mutant and wild type) after AuNPs treatment (Figure 4D and Figure S15). These data are congruent with previous studies concerning the role of mutant p53 on cancer cell proliferation and highlight the potential use of these nanostructures as a personalized therapy in cancers bearing mutant p53. The efficacy of such antisense sequences was also confirmed through liposomal transfection of mutant and wild-type p53 cancer cells, as described in material and methods (Figures S16A–D and S17).

### 3.6. Modified Gold Nanoparticles Reduce Chemoresistance in Mutant p53 Cancer Cells

**Figure 5:**
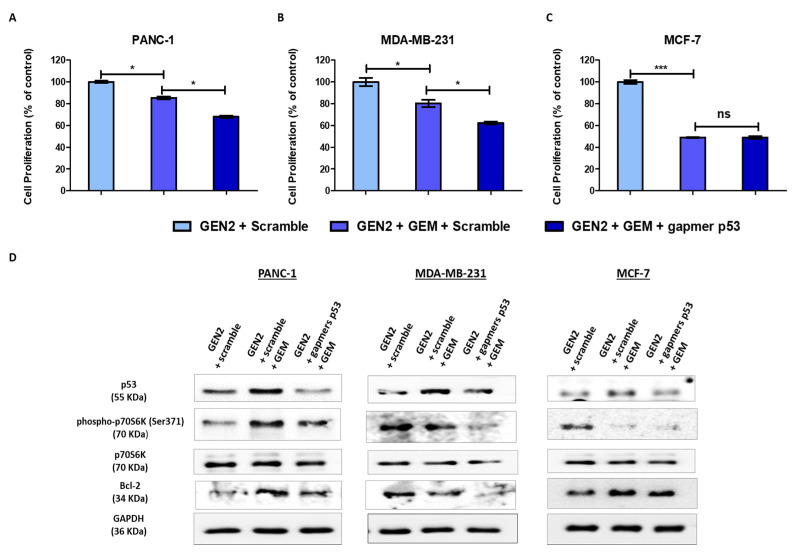
Modified gold nanoparticles reduce chemoresistance in mutant p53 cancer cells. (**A**–**C**) The indicated cells lines were seeded in 96-well plates, incubated overnight, and treated with GEN2-AuNPs functionalized with 120 pmoles of gapmers against p53. Then, 5 h after the treatment, the cells were treated with GEM (4.5 µM) incubated for 72 h. At the end of treatment, their viability was assessed. The values of treated cells were normalized to that of untreated controls and reported as mean ± ES. (**D**) The cells lines were seeded in 12-mm diameter culture dishes, incubated overnight, and treated with GEM (4.5 µM) for 72 h. Whole-cell extracts were processed for Western blot analysis of the indicated antibodies. GAPDH protein level in the same extract was used as a control loading. Statistical analysis was performed using one-way ANOVA (each group vs. control). (*** *p* < 0.001, * *p* < 0.05).

Then, the cell viability was studied in both mutant and wild-type p53 cancer cell lines when the nanostructures functionalized with gapmers targeting TP53 were combined with GEM treatment. Interestingly, in PANC-1 and MDA-MB-231 mutant p53 cancer cells, the combination of the functionalized nanostructures with GEM overcame the chemoresistance to GEM, leading to an enhancement of the cytotoxic effect (Figure 5A,B). In the case of MCF-7, the nanomaterials functionalized with p53-gapmers did not affect cell viability when employed in combination with GEM (Figure 5C). These data strongly suggest that oncogenic mutant p53 proteins confer chemoresistance to gemcitabine and that selective therapeutic targeting of mutant p53 can enhance the effect of chemotherapy. The beneficial effect of such antisense sequences combined with GEM was also confirmed through liposomal transfection of mutant and wild-type p53 cancer cells, as described in material and methods (Figure S18).

To elucidate the mechanism underlying the therapeutic effect of such nanostructures, we assessed the levels of some critical markers related to apoptosis and cell growth after their incubation with GEM in mutant and wild-type p53 cancer cells (Figure 5D and Figure S19).

After GEM treatment, mTOR signaling was enhanced in PANC-1 pancreas cancer cells and was not affected in MDA-MB-231 breast cancer cells, thus contributing to chemoresistance. Interestingly, the combined treatment with functionalized nanostructures targeting mutant p53 reduced mTOR signaling in such mutant p53-associated cancer cells. Moreover, the protein levels of the antiapoptotic protein Bcl-2 also decreased in PANC-1 and MDA-MB-231 after combined treatment, suggesting the activation of the apoptotic program to overcome chemoresistance to GEM. On the other side, mTOR signaling and Bcl-2 protein were not affected in MCF-7 cells after combined treatment. These data revealed the ability of these functionalized nanoparticles to reduce chemoresistance in mutant p53 cancer cells through the modulation of signaling pathways involved in cell growth and apoptosis.

## 4. Discussion

Nowadays, many clinical trials have failed to demonstrate an improvement in the overall survival of cancer patients treated with GEM in combination with different anticancer drugs. Extensive research is currently focused on identifying novel potential therapeutic targets to overcome GEM resistance in different cancers [65,66,67]. In this regard, strategies based on mTOR inhibition and/or AMPK activation have been shown to sensitize pancreatic cancer cells to GEM [68,69].

The p53 tumor suppressor protein is a master transcriptional regulator that controls several critical physiological pathways, such as cell cycle arrest, apoptosis, senescence, DNA damage response, and metabolism [1].

It is emerging that mutant p53 proteins, contrarily to their wild-type p53 counterpart, reduce the response of cancer cells to chemotherapy conferring chemoresistance to DNA damaging agents. This mainly occurs through a sophisticated dysregulation of cellular signaling pathways and by modulating the activity of transcription factors related to autophagy regulation, mitochondria biogenesis, and stress metabolic adaptation of cancer cells [10,70,71,72,73]. Hence, novel approaches aimed to inhibit the expression and function of oncogenic mutant p53 proteins represent a valid therapeutic approach to overcome cancer chemoresistance.

The functionalization of the gold nanoparticles with different agents allows taking advantage of their properties without producing adverse side effects. Among the various chemical modifications existing, one of the more exploited is PEG. This molecule stabilizes the electrostatic proprieties of AuNPs and reduces the instability produced at the time to conjugate the branched polyethylenimine (bPEI), which originates due to the large difference of charges between them. Furthermore, the use of short bPEI helps to reduce its toxicity in cell lines [74]. The functionalization of AuNPs with both agents leads to stable and positive charged nanoparticles.

In this research, an innovative approach to increase the potential of the particles and their transfection efficacy has been designed. Instead of replacing the molecule of bPEI for a larger one, which would increase the overall toxicity, we introduced to the formulation a second molecule of low molecular bPEI, previously modified with an SPDP crosslinker. This way, the length of the polymer can be controlled and tune the properties of the nanostructure, providing a high concentration of positive charges. This may be due to a more efficient electrostatic charge packaging, translating into a bigger transfection capacity. Another advantage is the kind of bond created in the process, a disulfide bond, which is considered a stimulus-sensitive linker. This one is sensitive to reduction stimulus; thus, in the presence of a tumoral environment, where the concentration of reducing agents is higher, 85% of the polymer would be released along with its therapeutic cargo.

To maximize the binding capacity of the multifunctional AuNPs with oligonucleotides, three factors have been optimized. The first one is the molar ratio of bPEI/oligonucleotide, which has been determined that at 8 (N/P ratio 34) provides a great binding in comparison with PEI of higher MW at the same conditions [59]. The second one is the use of an agent to reduce the pH of the nanoparticles’ dispersion to increase the positive concentration of the bPEI. A citrate buffer solution provided an adequate pH reduction without destabilizing or degrading the nanoparticles or their components, and at 3.5 pH, displayed better binding results due to most of the amino groups may be protonated. The third factor is the concentration of the buffer itself, showing that higher concentrations of it can disfavor the binding.

Then, we assessed the anticancer effect of modified AuNPs functionalized with therapeutic gapmers targeting mutp53 proteins in pancreatic and breast cancer cell lines (Table 3). Firstly, we observed that our modified nanostructures could reduce the levels of p53 proteins in a panel of cancer cells. Interestingly, the cell proliferation was affected exclusively in cancer cells carrying the mutp53 proteins (PANC-1 and MDA-MB-231), but not the wild-type counterpart (MCF-7). These data are consistent with the pro-survival role that mutant p53 plays in cancer cells, especially through the establishment of a mutant p53/NF-Y protein complex which leads to an aberrant cell cycle transcriptional regulation [9]. These observations suggest that our nanoparticles are safe in not-tumoral cellular models carrying wild-type p53 and can be exploited for the treatment of cancer cells bearing mutant p53 proteins, expanding their potential in in vivo applications.

Later, we also evaluated the effect of GEM treatment in cancer cell lines bearing mutant p53 or wild-type proteins. Interestingly, we observed that this standard chemotherapeutic leads to an increase in both wild-type and mutp53 protein levels. It is tempting to speculate that this response may lead to different effects on cancer cell proliferation and apoptosis depending on TP53 mutational status. In this regard, especially in PANC-1, GEM increased the activity of the mTOR pathway, which we detected through the phosphorylation of its downstream target p70S6 kinase. In addition, the chemotherapy also augmented the levels of Bcl-2 protein, which is a classical inhibitor of apoptosis and represents a target for cancer therapy [63]. However, in MDA-MB-231 cancer cells, these molecular markers were not affected by GEM treatment, and, in contrast, they were strongly reduced in wild-type p53 MCF-7 cells. This data strongly indicates that the different modulation of Bcl-2/p70S6K pro-tumoral axis may be linked to the different p53 status in cancer cells and that, in the case of PANC-1, the induction of Bcl-2 may partially explain their drug resistance phenotype.

Finally, we assessed whether our nanostructures, through their targeting of p53 proteins, could revert the acquired chemoresistance typical of certain mutp53 cancer cells and therefore represent a personalized treatment for cancer patients with alterations in the TP53 gene. Remarkably, these nanomaterials were able to increase the activity of GEM in PANC-1 and MDA-MB-231 cancer cell lines but not in MCF-7 cancer cells. In this regard, in wild-type p53 cells, the combined therapy did not enhance the effect of GEM, and the reduction observed in their viability was only due to GEM toxicity. As mentioned above, the drastic difference between the cancer cell lines carrying mutant p53 proteins versus the wild-type model may be explained by, among other causes, the opposite effects that p53 variants play in the context of cancer cells proliferation and chemoresistance. One possible explanation for which cancer cells with mutp53 are more resistant to chemotherapeutic agents could be related to PUMA-induced apoptosis and p21-mediated cell cycle arrest [75].

Later, we investigated the molecular mechanisms underlying the activity of our AuNPs in mutant p53 cancer cells. We observed that these nanostructures were able to inhibit the mTOR signaling pathway and the antiapoptotic protein Bcl-2, thus overcoming drug resistance.

Overall, these data allow us to speculate that both apoptosis induction and mTOR inhibition, resulting after combined therapy, might contribute to the reduction of cell viability observed in mutant p53 cancer cells. The data presented herein provide new therapeutic options based on the delivery of modified oligonucleotides through modified nanomaterials in overcoming cancer resistance in apoptosis-refractory tumors bearing mutant p53 cancer cells.

## Figures and Tables

**Table 1:** Functionalization agents and amount used for use in AuNPs.

GEN	LP-PEG	LP-PEI	Traut’s Reagent	PDP-PEI
1	3 nmol	7 nmol	-	-
2	3 nmol	7 nmol	40 nmol	40 nmol

**Table 2:** Oligonucleotides used in this study.

Entry	Oligonucleotide	Sequence
1	Control 1	5′-**ACGUG**ACACGTTCGGA**GAAUU**-3′
2	Control 2	5′-**UGCGC**TCCTGGACGT**AGCCU**-3′
3	Gapmer p53.1	5′-**CAAAG**CTGTTCCGTC**CCAGU**-3′
4	Gapmer p53.2	5′-**GACUC**CAGTGGTAA**TCTAC**-3′
5	Gapmer p53.3	5′-**GAAAU**TTGCGTGTG**GAGUA**-3′
6	Gapmer p53.4	5′-**GGACA**TACCAGCTTAG**AUUUU**-3′

**Table 3:** p53 status of cell lines used in this study.

Entry	Cell Line	Tumor Tissue	P53 Mutation
1	PANC-1	Pancreas	R273H
2	MDA-MB-231	Breast	R280K
3	MCF-7	Breast	Wild Type

## Data Availability

The data are contained within the article. The raw data of Western blots study are available on request from the corresponding authors.

## Supplementary Materials

The following are available online at https://www.mdpi.com/article/10.3390/pharmaceutics13122067/s1, Figure S1: TEM image of non-functionalized AuNPs, Figure S2: DLS, hydrodynamic size and zeta potential measurements, Figure S3: Study of zeta potential and size comparison, Figure S4: Cytotoxicity assay of the modified bPEIs and AuNPs, Figure S5: Pyridin-2-thione quantification, Figure S6: Size and Zeta Potential comparison of GEN1, GEN2, and GEN3, Figure S7: Size and Zeta Potential measurements at different times, Figure S8: GEN2-AuNPs gel retardation assay and quantification, Figure S9: Cell viability studies in cancer cells after GEN2-AuNPs treatment, Figure S10: Fluorescence studies of oligonucleotide polyT(10)FAM release from GEN2-AuNPs in MCF-7, Figure S11: Fluorescence studies of oligonucleotide polyT(10)FAM using Lipofectamine 2000 in PANC-1, Figure S12: Fluorescence studies of oligonucleotide polyT(10)FAM using Lipofectamine 2000 in MCF-7, Figure S13: Mutant and wild-type p53 cancer cells show different sensitivity to chemotherapy, Figure S14: Quantitative analysis of p53/GAPDH, phospho (Ser371)-p70S6K/GAPDH, p70S6K/GAPDH, and Bcl-2/GAPDH ratios, Figure S15: Quantitative analysis of p53/GAPDH ratios, Figure S16: Mutant p53 knock-down reduces cancer cell proliferation, Figure S17: Quantitative analysis of p53/GAPDH ratios, Figure S18: Gapmers targeting mutant p53 overcome chemoresistance to GEM, Figure S19: Quantitative analysis of p53/GAPDH, phospho(Ser371)-p70S6K/GAPDH, p70S6K/GAPDH, and Bcl-2/GAPDH ratios. Instrumental analysis and Methods: General information, TEM, Dynamic light scattering (DLS) and Zeta-Potential, Gel retardation assay, Alamar Blue Viability Assay, Western blot analysis, Fluorescence of polyT(10)FAM, Statistical analysis. Synthesis of linkers and polymer derivatives: 2,5-dioxopyrrolidin-1-yl 5-(1,2-dithiolan-3-yl)pentanoate (Lipoic activated ester linker), (3-(pyridin-2-yldisulfanyl) propanoic acid), 2,5-dioxopyrrolidin-1-yl 3-(pyridin-2-yldisulfanyl)propanoate (SPDP linker), Synthesis of LP-PEI (2000 MW), Synthesis of SPDP-PEI (2000 MW), Synthesis of LP-PEG (3000 MW).
