# Supplementary material for: Modified Gold Nanoparticles to Overcome the Chemoresistance to Gemcitabine in Mutant p53 Cancer Cells
Source: Pharmaceutics. 2021 Dec 3;13(12):2067. doi: 10.3390/pharmaceutics13122067 (PMC8703659; doi:10.3390/pharmaceutics13122067)
Supplement: Supplementary file 1 [file pharmaceutics-13-02067-s001.zip › pharmaceutics-1440468-supplementary.pdf]

pharmaceutics

MDPI

Supplementary Materials: Modified Gold Nanoparticles to Overcome the Chemoresistance to Gemcitabine in Mutant p53 Cancer Cells

Eduardo García-Garrido, Marco Cordani, Álvaro Somoza

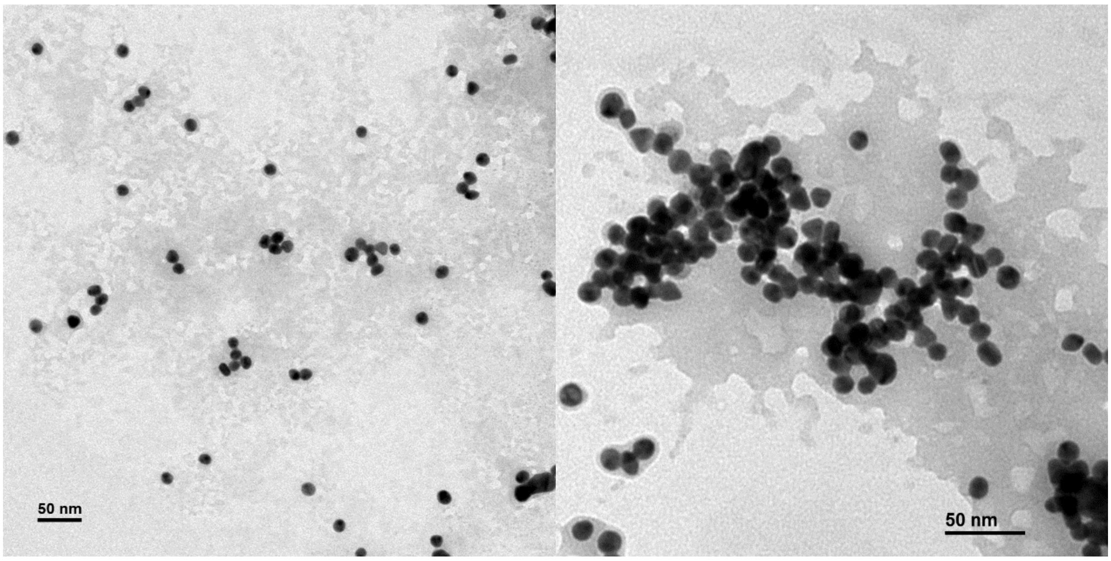

Size Aimed	Method	Number of measures	Average values
13 nm	Turkevitch's	47	12.98 ± 1.5 nm

Figure S1. TEM images of AuNPs synthesized by the Turkevitch's method. Images are taken at 50 and 150k, respectively. The average size has been obtained evaluating the area of 47 nanoparticles of the first image with the software Fiji-ImageJ.

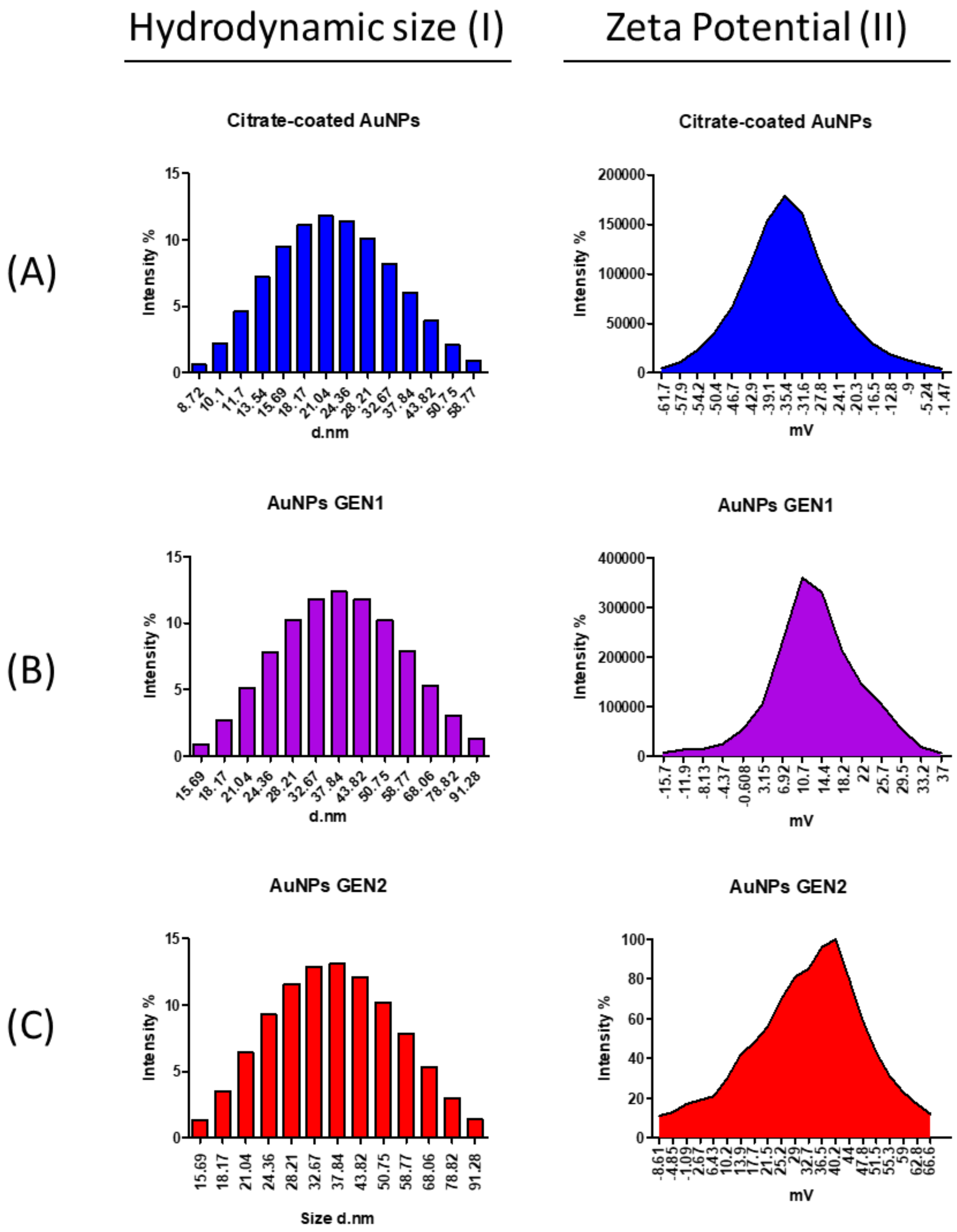

Figure S2. DLS, hydrodynamic size (I) and zeta potential (II), measurements of citrate coated AuNPs (**A**), GEN1 AuNPs (**B**) and GEN2 AuNPs (**C**).

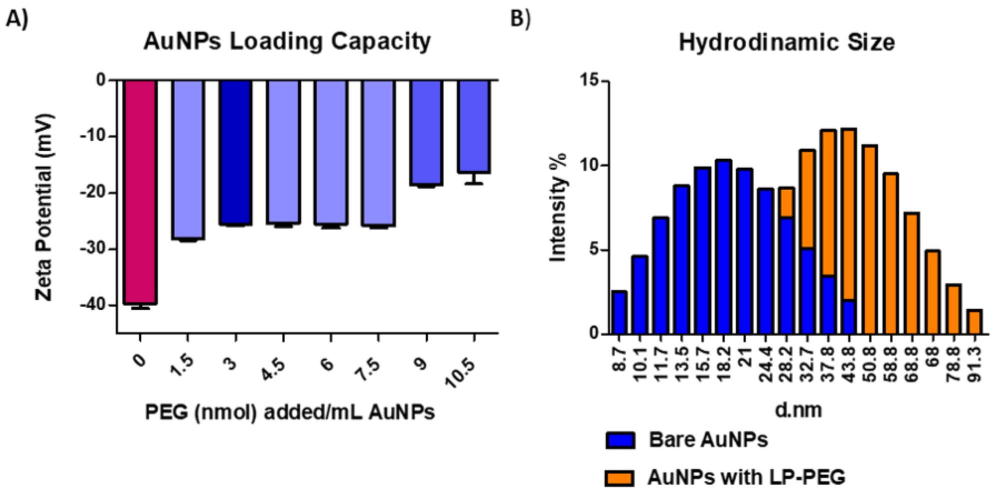

Figure S3. (**A**) Study of zeta potential changes adding increasing amounts of LP-PEG. It was determined that after the addition of 10,000 pmols the potential kept constant and that less of 3000 pmols the nanoparticles were not stable enough. (**B**) Size comparison of AuNPs before and after being functionalized only with LP-PEG.

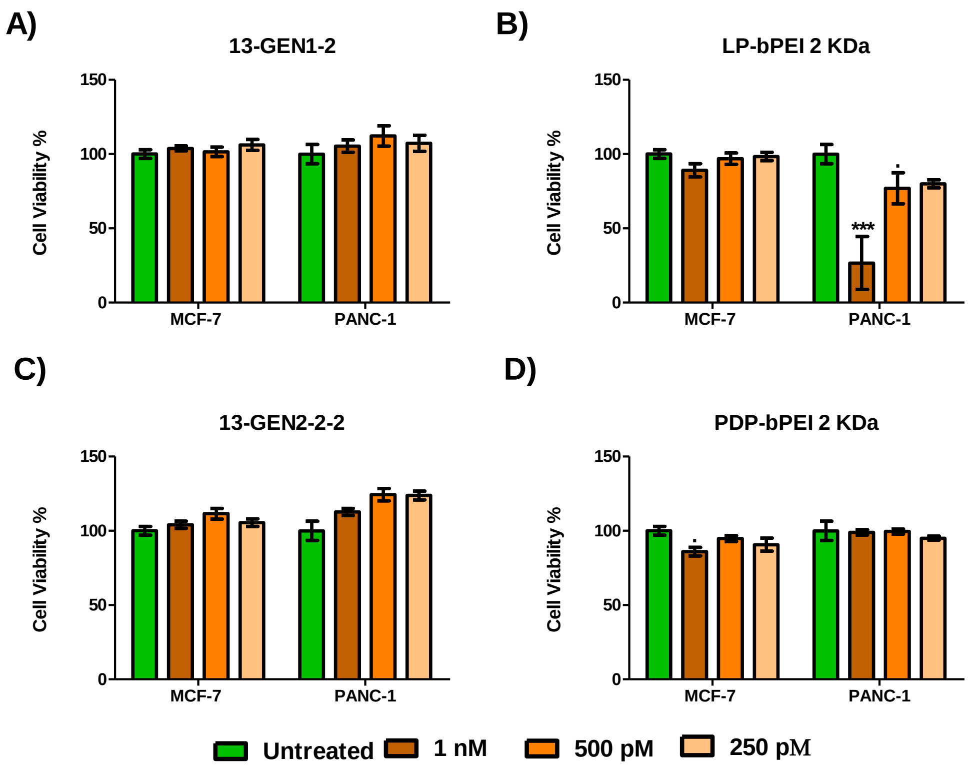

Figure S4. Cytotoxicity assay of the modified bPEIs (LP-PEI and SPDP-PEI) (**B**–**D**) used in the functionalization of the AuNPs and two types of functionalized AuNPs evaluated (GEN1 and GEN2) (**A**–**C**), at concentrations of 1, 0.5 and 0.25 nM in cancer cell lines MCF7 and PANC1. The amount of LP-bPEI used were 875, 437.5 and 218.75 pmol, and for PDP-bPEI were 5950, 2975 and 1487.5 pmol, the same amount as that present on the particles at the concentrations tested.

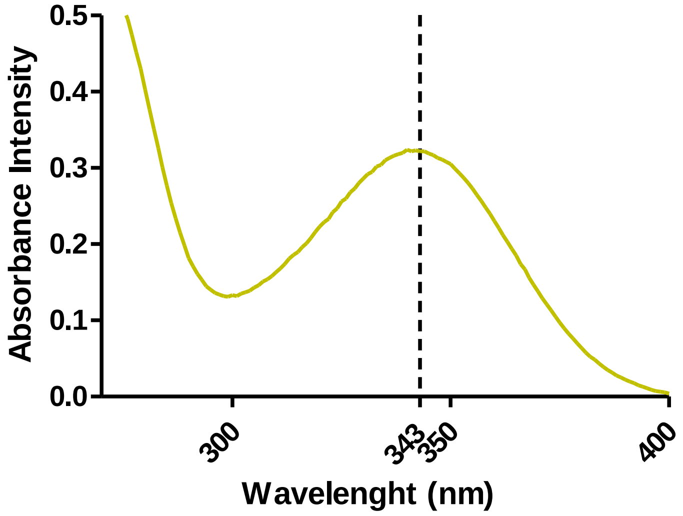

Figure S5. Pyridin-2-thione quantification. The molecule is released during the functionalization of 13-GEN1-2 AuNPs with PDP-bPEI 2 KDa. Through the Beer-Lambert law, the molecule released was quantified (41.65 nmol).

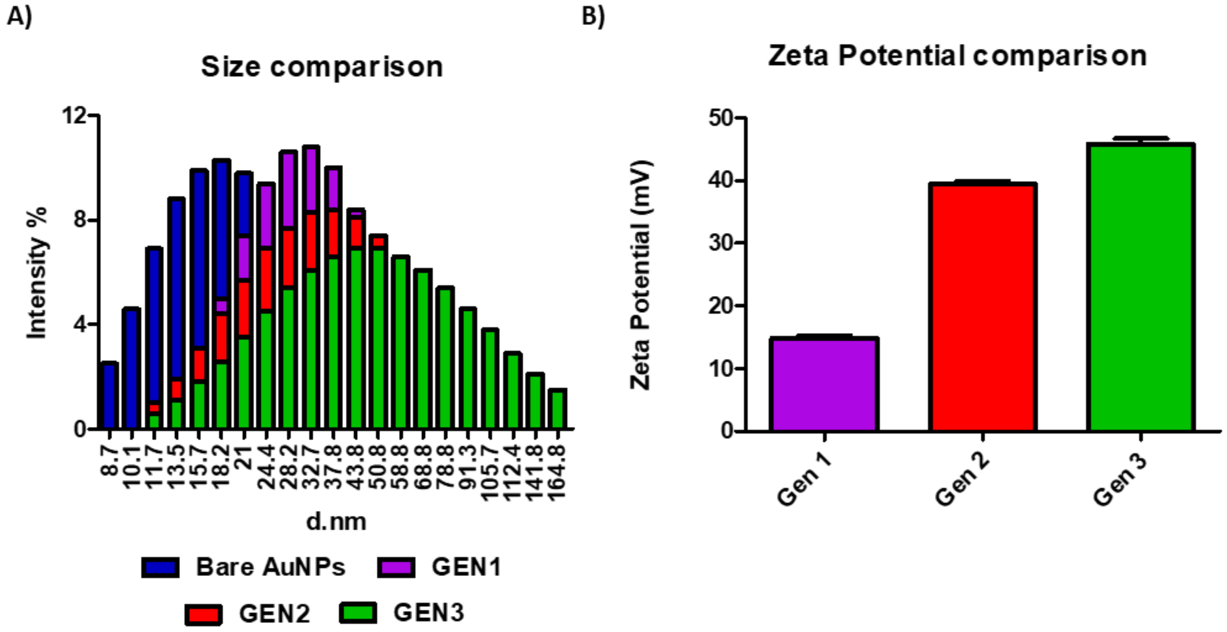

Figure S6. Size (**A**) and Zeta Potential (**B**) comparison of GEN1, GEN2 and GEN3. Gen3 shows a wider size distribution due to lower functionalization and low stability, and a slightly increase of potential which leaded to discard these AuNPs for practical use.

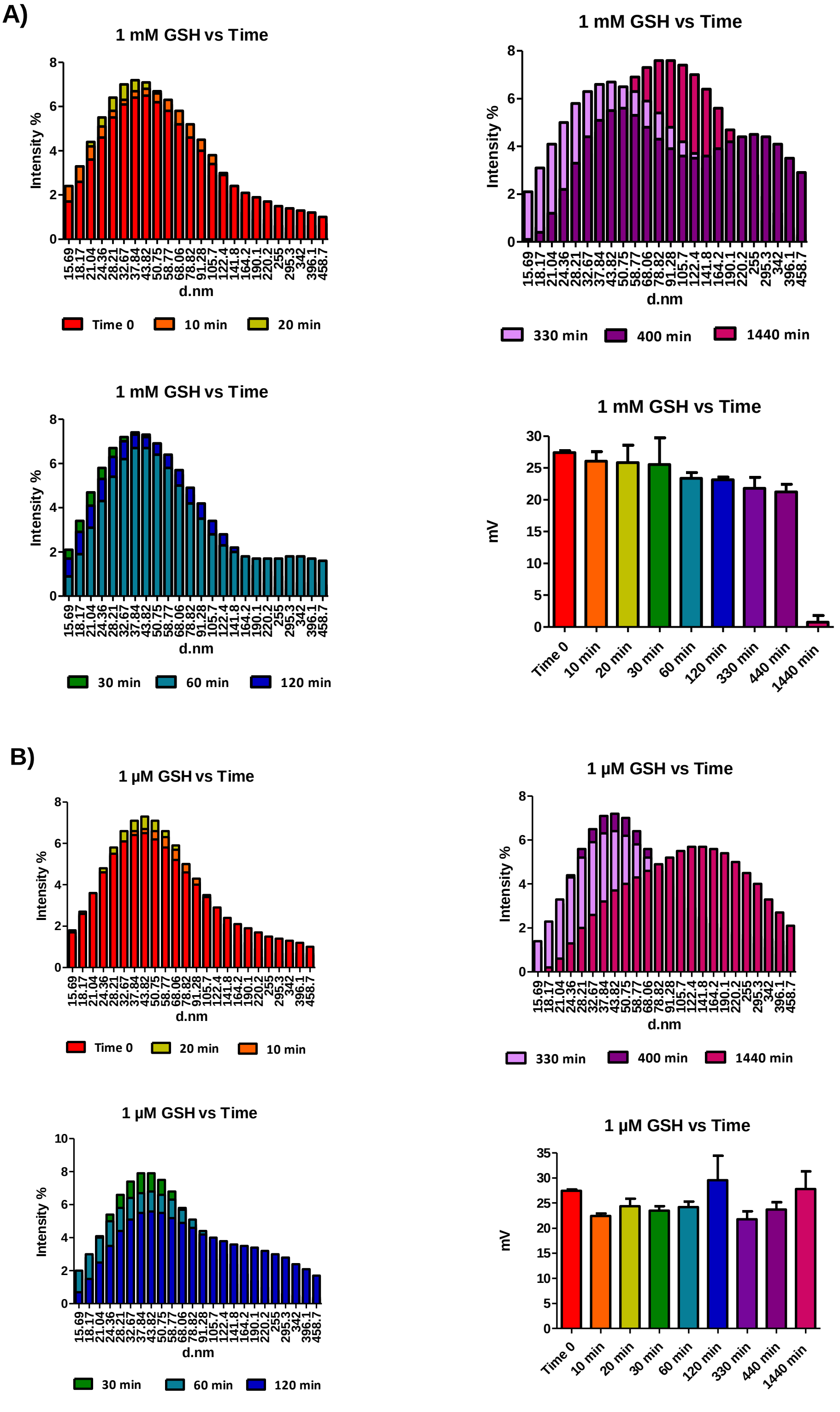

Figure S7. Size and Zeta Potential measurements at 10, 20, 30, 60, 120, 330, 400 min and 24 h of GEN2-AuNPs in presence of 1 mM (**A**) and (**B**) 1 µM of GSH. It can be observed significant changes in zeta-potential at 24 h after the incubation of 1 mM of GSH, which indicates the loss of bPEI chains.

(**A**)

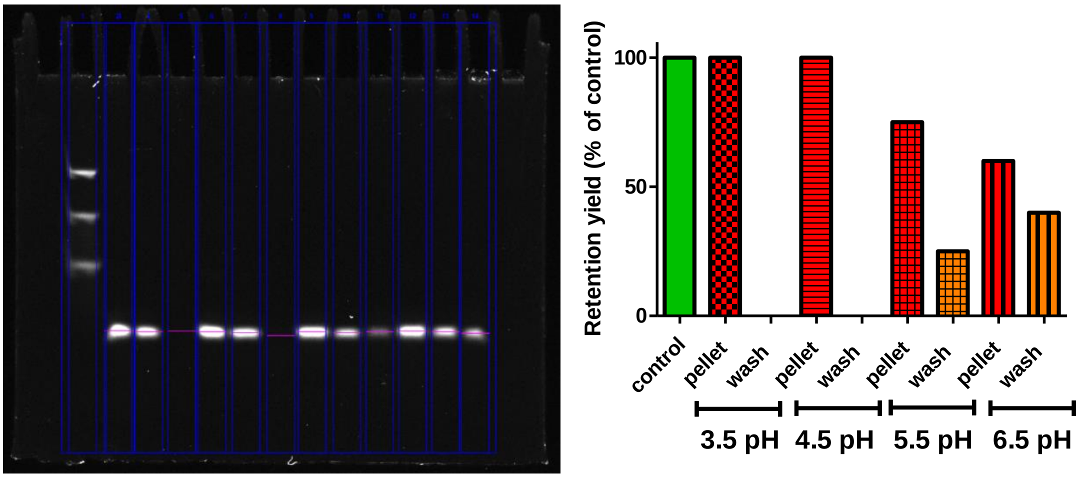

pH	Pellet % (oligonucleotide bonded)	Wash %(oligonucleotide not-bonded)
6.5	60	40
5.5	75	25
4.5	100	0
3.5	100	0

(**B**)

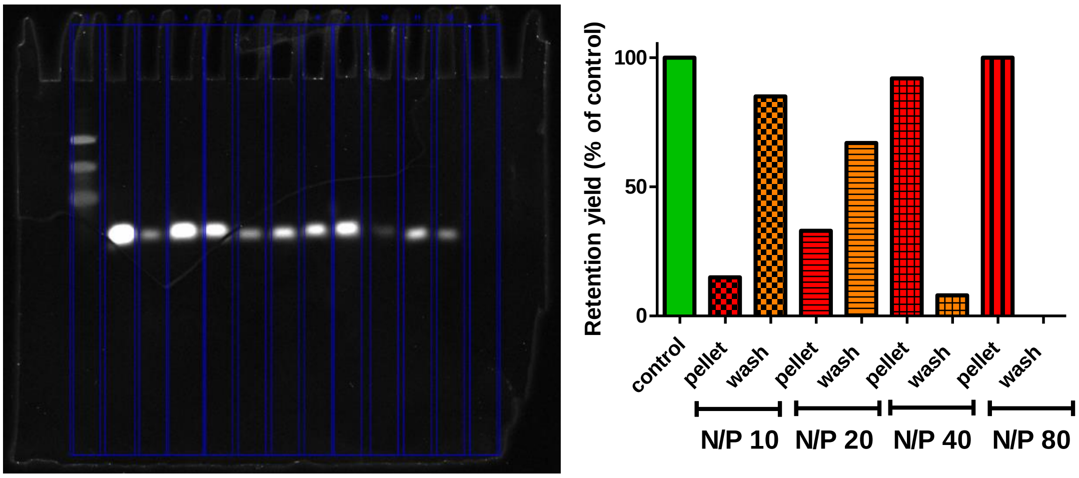

N/P	Pellet % (oligonucleotide bonded)	Wash % (oligonucleotide not-bonded)
80	100	0
40	92	8
20	33	67
10	15	85

(C)

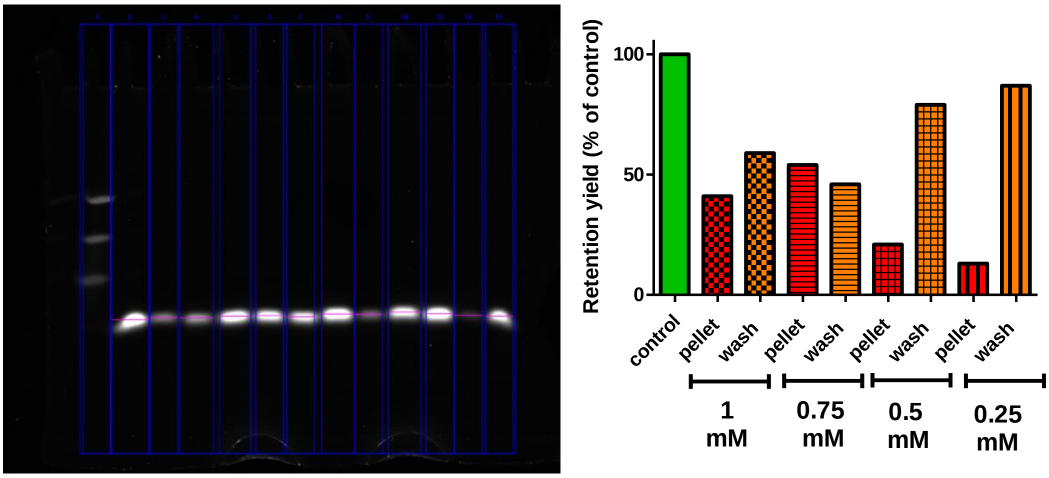

Buffer (mM)	Pellet % (oligonucleotide bonded)	Wash % (oligonucleotide not-bonded)
1	41	59
0.75	54	46
0.5	21	79
0.25	13	87

Figure S8. GEN2-AuNPs gel retardation assay and quantification to test the optimal bPEI/polyT(10)FAM binding conditions. (**A**) pH study. AuNPs variable pH of 3.5, 4.5 and 5.5. Fixed buffer concentration to 50 mM and bPEI/PolyT(10)FAM N/P ratios of 80. (**B**) Molar ratio study. Variable bPEI/PolyT(10)FAM N/P ratios 10, 20, 40 and 80. Fixed pH at 3.5 and buffer concentration 1 mM. (**C**) Buffer concentration study. Variable buffer concentrations of 1, 0.75, 0.50 and 0.25 mM. Fixed pH at 3.5 and bPEI/PolyT(10)FAM molar ratios of 20.

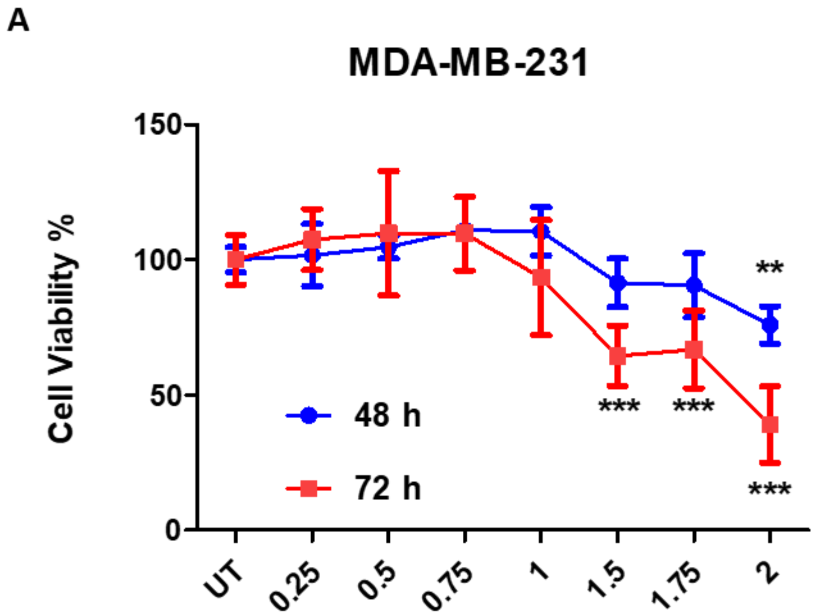

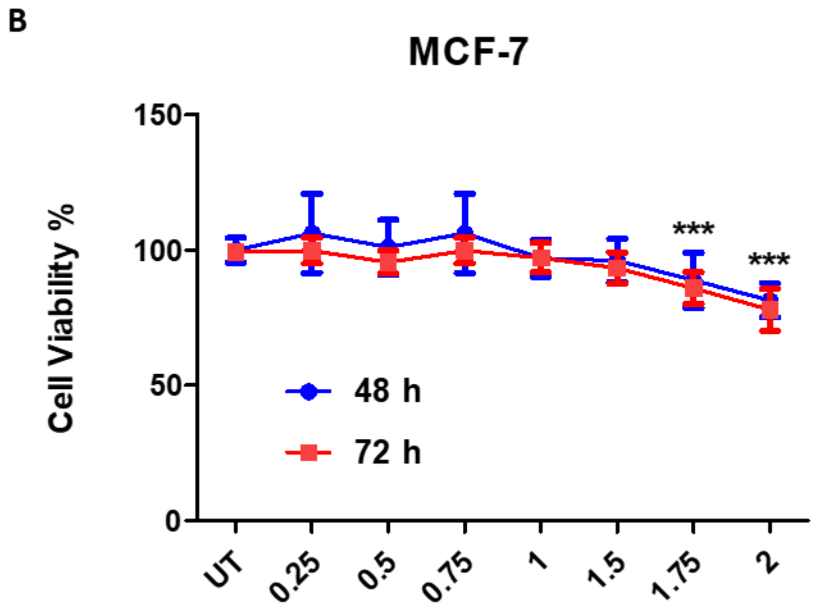

Figure S9. Cell viability studies in cancer cells after GEN2-AuNPs treatment. (**A**) MDA-MB-231 and (**B**) MCF-7 breast cancer cells were seeded in 96-well plates, incubated with GEN2-AuNPs for 48 and 72 h at the concentrations indicated, ranging from 0.25 to 2 nM. The values of treated cells were normalized to that of untreated controls and reported as mean ± SE. Statistical analysis was performed using one-way ANOVA (each group vs. control). (*** $p < 0.001$, ** $p < 0.01$).

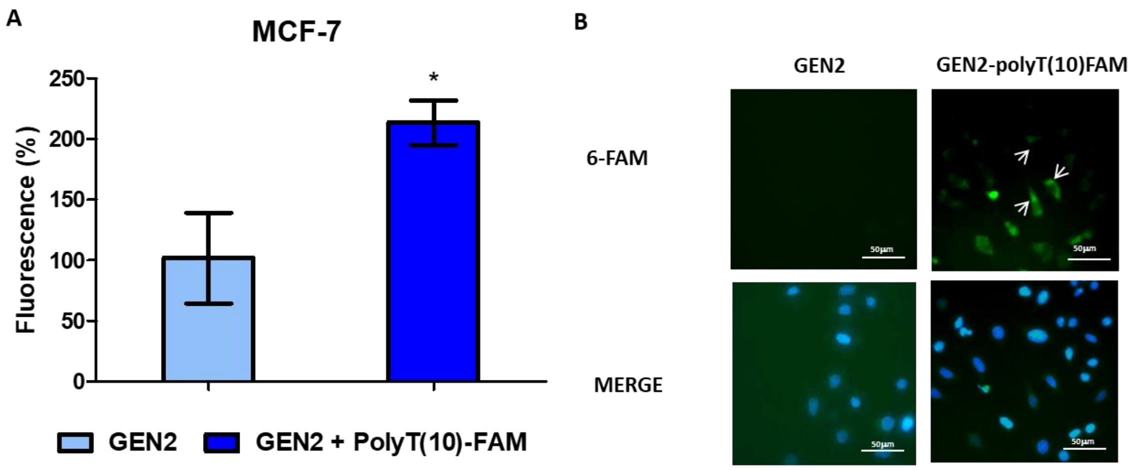

Figure S10. Fluorescence studies of oligonucleotide polyT(10)FAM release from GEN2-AuNPs. (**A**) The MCF-7 breast cancer cells were incubated with GEN2-AuNPs-polyT(10)FAM for 24 h. The fluorescence was measured with a multiplate reader and normalized to that of controls (GEN2) and reported as mean ± SD. (**B**) Representative fluorescence images of MCF-7 cancer cells treated with GEN2 and treated with GEN2-polyT(10)FAM. FAM in green and nucleus are labeled in blue by Hoechst staining. Statistical analysis was performed using one-way ANOVA (each group vs. control). (* $p < 0.05$**).**

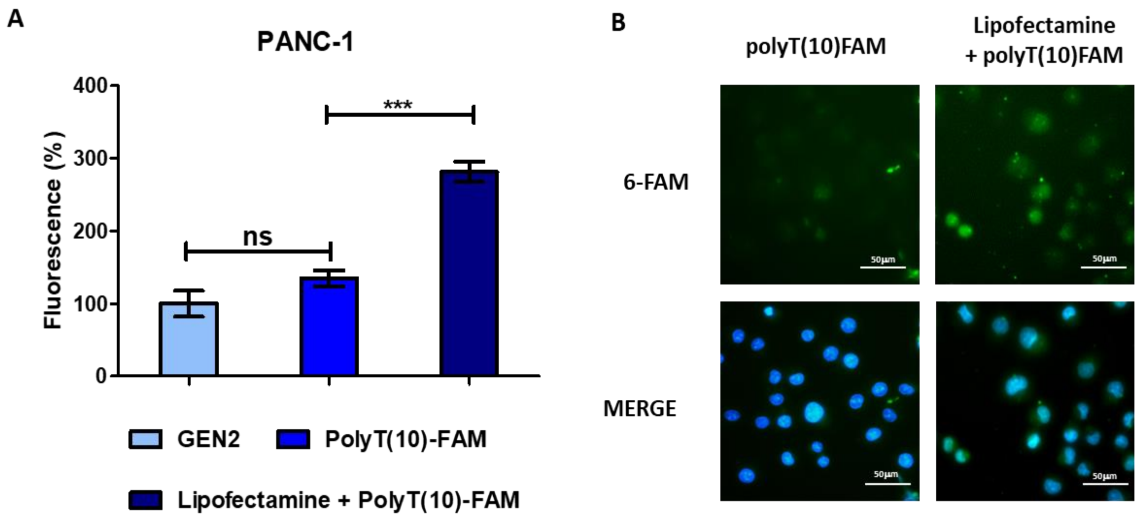

Figure S11. Fluorescence studies of oligonucleotide polyT(10)FAM using Lipofectamine 2000. (**A**) The PANC-1 pancreas cancer cells were incubated with GEN2, polyT(10)FAM and transfected with 100 pmoles polyT(10)FAM using Lipofectamine 2000 for 24 h. The fluorescence was measured with a multiplate reader and normalized to that of controls (GEN2) and reported as mean ± SD. (**B**) Representative fluorescence images of PANC-1 cancer cells treated with polyT(10)FAM and transfected using Lipofectamine 2000. FAM in green and nucleus are labeled in blue by Hoechst staining. Statistical analysis was performed using one-way ANOVA (each group vs. control). (*** $p < 0.001$**).**

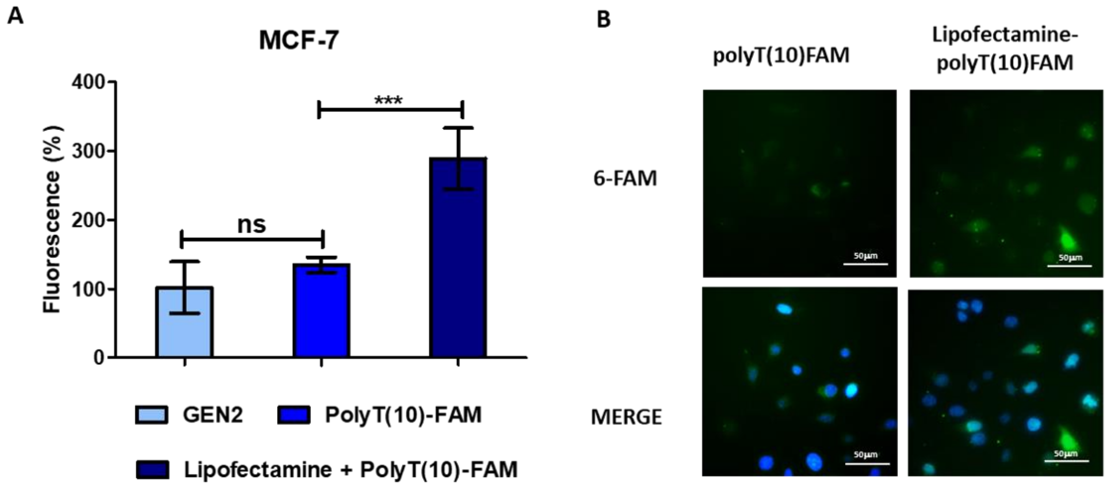

Figure S12. Fluorescence studies of oligonucleotide polyT(10)FAM using Lipofectamine 2000. (**A**) The MCF-7 breast cancer cells were incubated with GEN2, polyT(10)FAM and transfected with 100 pmoles polyT(10)FAM using Lipofectamine 2000 for 24 h. The fluorescence was measured with a multiplate reader and normalized to that of controls (GEN2) and reported as mean ± SD. (**B**) Representative fluorescence images of MCF-7 cancer cells treated with polyT(10)FAM and transfected using Lipofectamine 2000. FAM in green and nucleus are labeled in blue by Hoechst staining. Statistical analysis was performed using one-way ANOVA (each group vs. control). (*** $p < 0.001$).

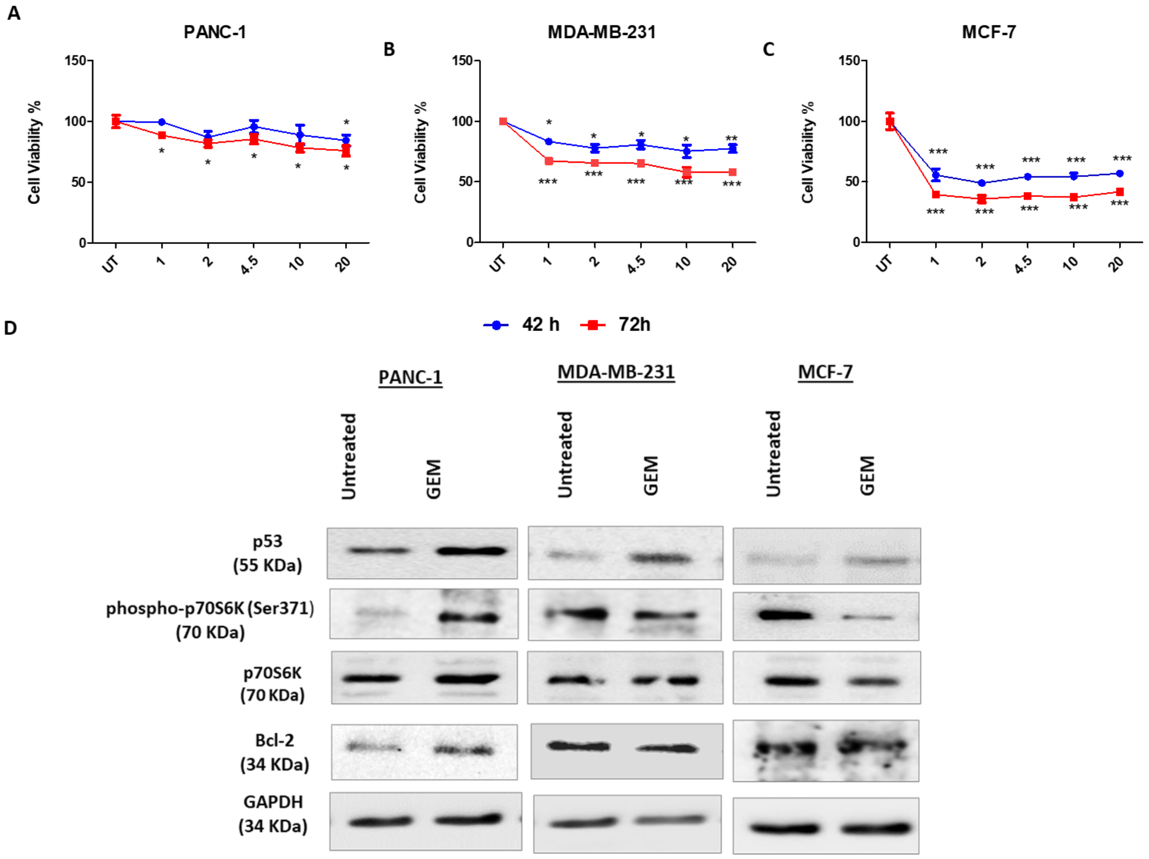

Figure S13. Mutant and wild-type p53 cancer cells show different sensitivity to chemotherapy. (**A**–**C**) PANC-1, MDA-MB-231 and MCF-7 pancreas and breast cancer cells were incubated with GEM at the indicated concentrations for 48 and 72 h and their viability assessed with the alamarBlue test. (**D**) The cells lines were seeded in 12-mm diameter culture dishes, incubated overnight, and treated with GEM (4.5 µM), for 72 h. Whole-cell extracts were processed for western blot analysis of the indicated antibodies. GAPDH protein level in the same extract was used as a control loading. The values of treated cells were normalized to that of untreated controls and reported as mean ± SD. Statistical analysis was performed using one-way ANOVA (each group vs. control). (*** $p < 0.001$; ** $p < 0.01$; * $p < 0.05$).

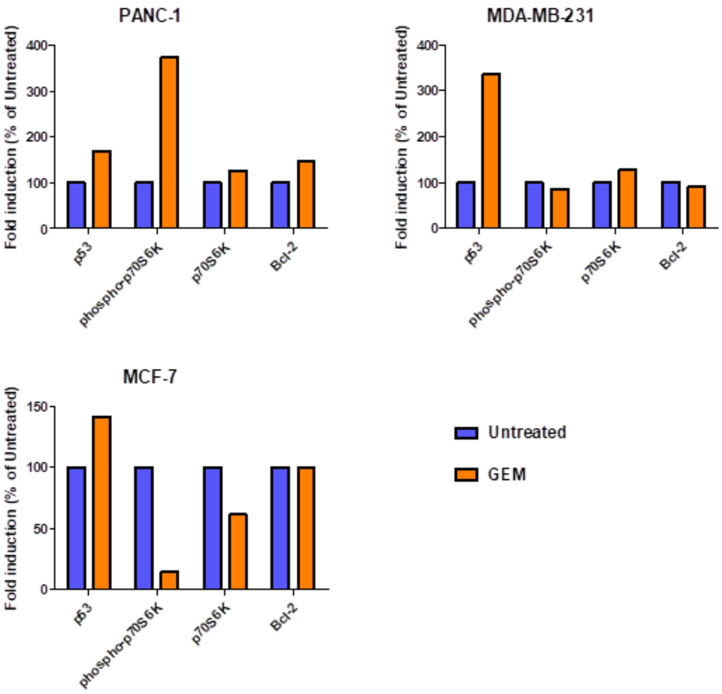

Figure S14. Quantitative analysis of p53/GAPDH, phospho (Ser371)-p70S6K/GAPDH, p70S6K/GAPDH and Bcl-2/GAPDH ratios. The bands shown in Figure S13D were scanned as digital peaks and the areas of the peaks were reported as fold induction in percentage respect to the untreated, as described in Material and Methods.

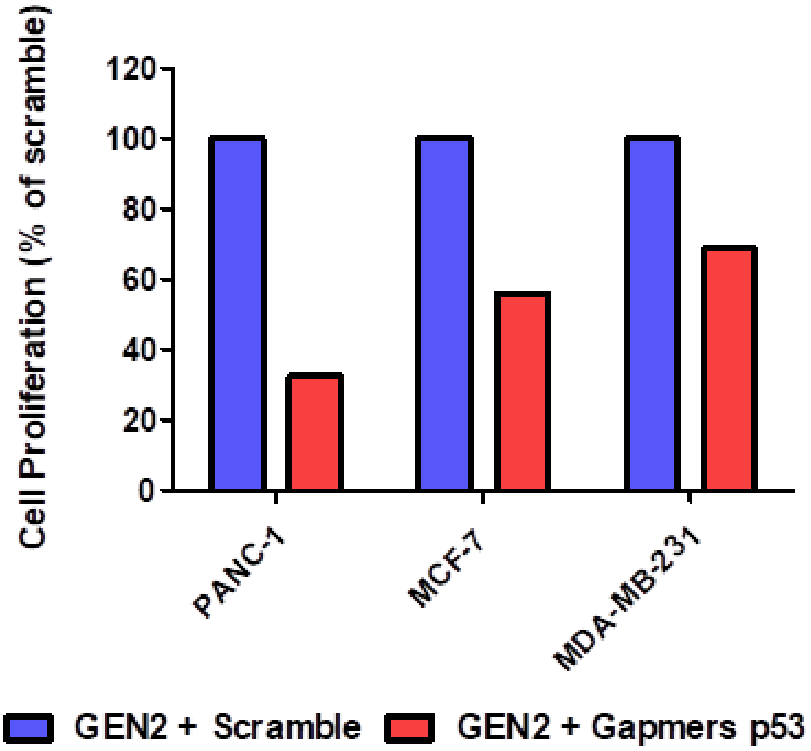

Figure S15. Quantitative analysis of p53/GAPDH ratios. The bands shown in Figure 4D were scanned as digital peaks and the areas of the peaks were reported as fold change in percentage with respect to the scramble, as described in Material and Methods.

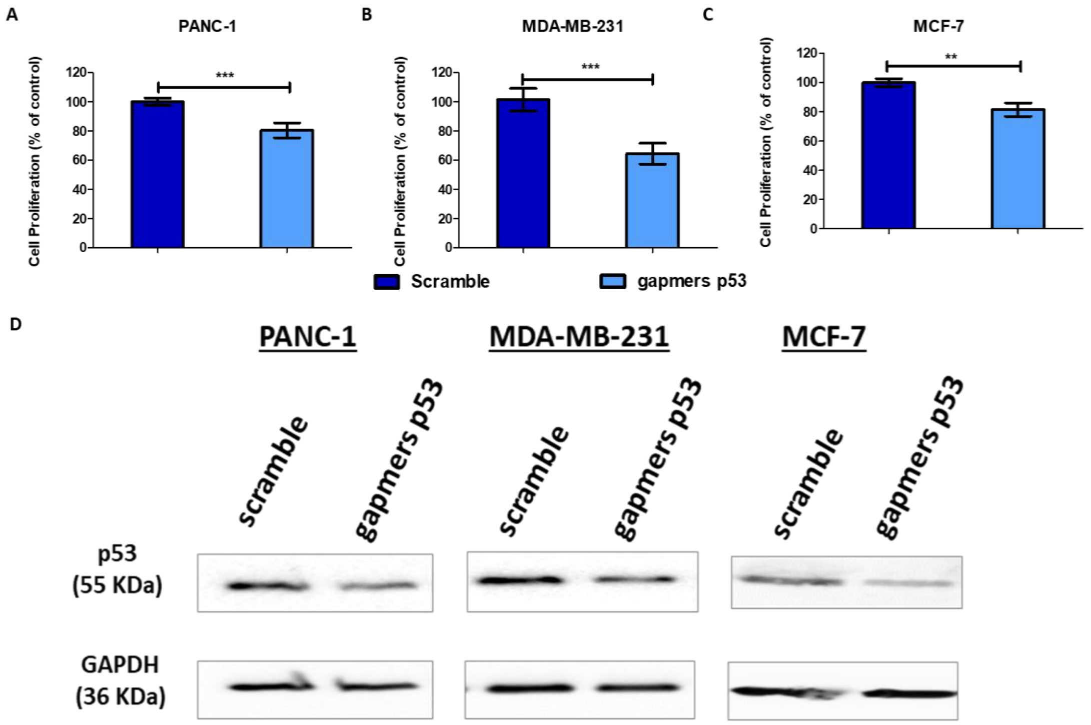

Figure S16. Mutant p53 knock-down reduces cancer cell proliferation. (**A**–**C**) The cells lines were seeded in 96-well plates, incubated overnight, and transfected with Lipofectamine 2000 mix containing 4 gapmers against p53. After 72 h, their viability was assessed with the alamarBlue test. (**D**) Whole-cell extracts were processed for western blot analysis of the indicated antibodies. GAPDH protein level in the same extract was used as a control loading. Statistical analysis was performed using one-way ANOVA (each group vs. control). (*** $p < 0.001$; ** $p < 0.01$).

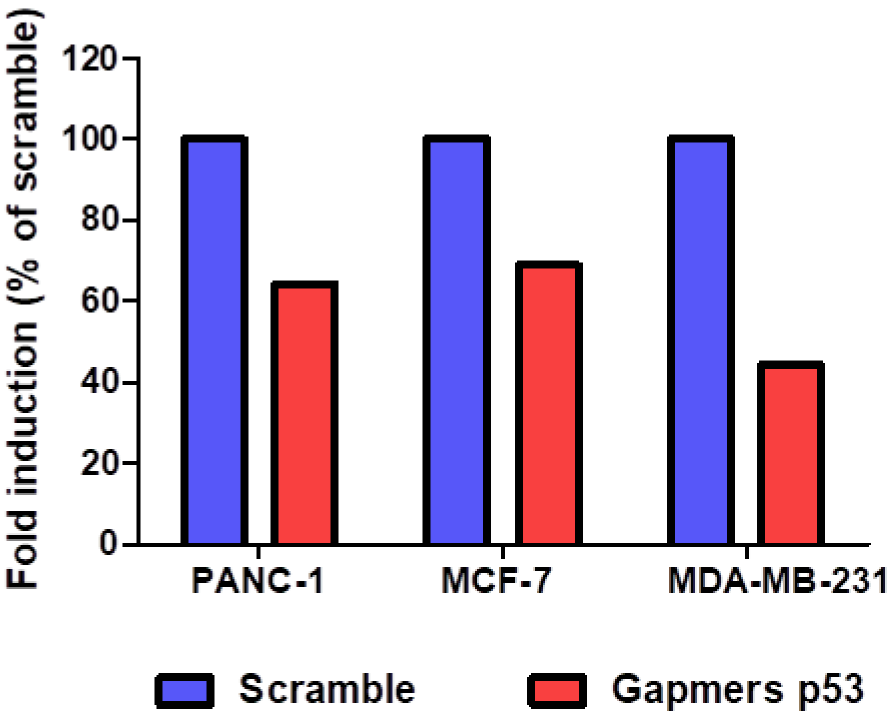

Figure S17. Quantitative analysis of p53/GAPDH ratios. The bands shown in Figure 15D 16D were scanned as digital peaks and the areas of the peaks were reported as fold change in percentage with respect to the scramble, as described in Material and Methods.

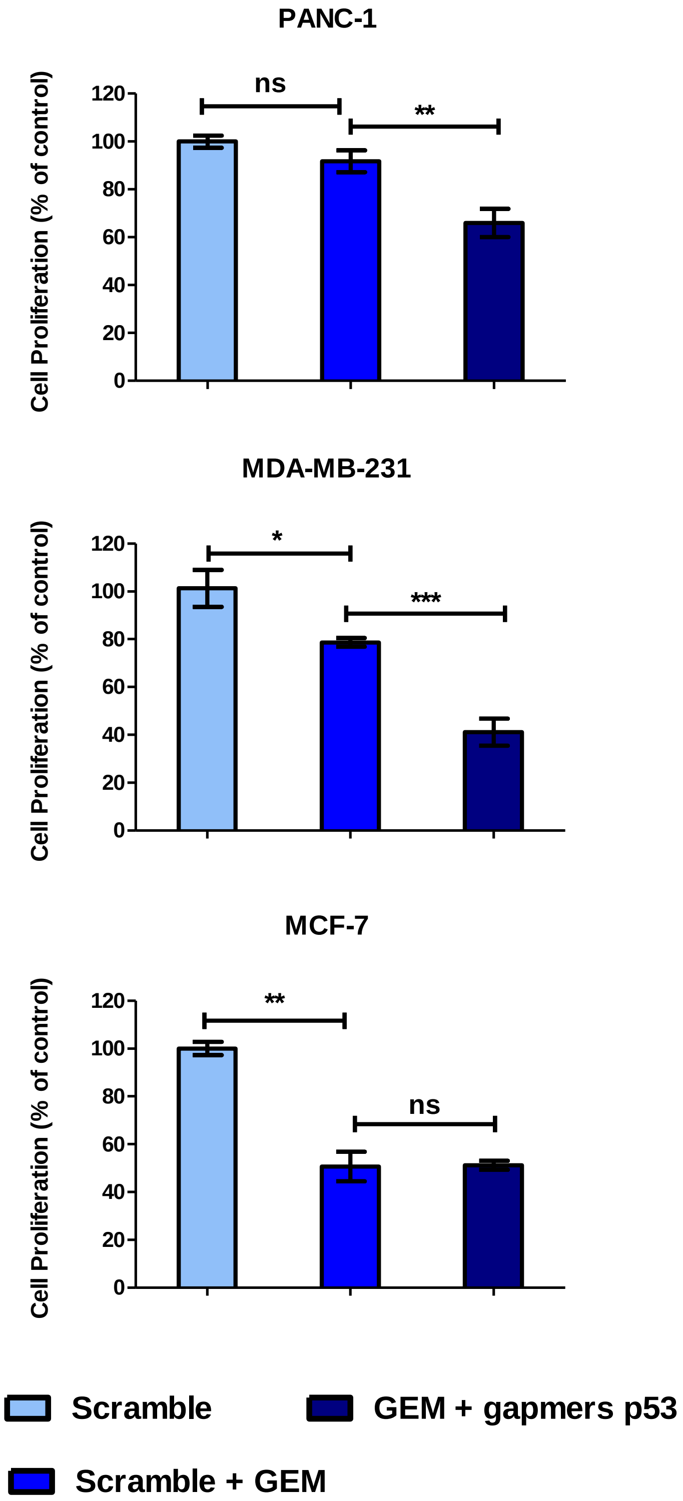

Figure S18. Gapmers targeting mutant p53 overcome chemoresistance to GEM. The indicated cells lines were seeded in 96-well plates, incubated overnight, and transfected with a mix containing 4 gapmers against p53. After 5 h from the transfection, the cells were treated with GEM (4.5 μM) incubated for 72 h. At the end of treatment, their viability was assessed with the alamarBlue test. The values of treated cells were normalized to that of untreated controls and reported as mean ± ES. Statistical analysis was performed using one-way ANOVA (each group vs. control). (*** $p < 0.001$; ** $p < 0.01$; * $p < 0.05$).

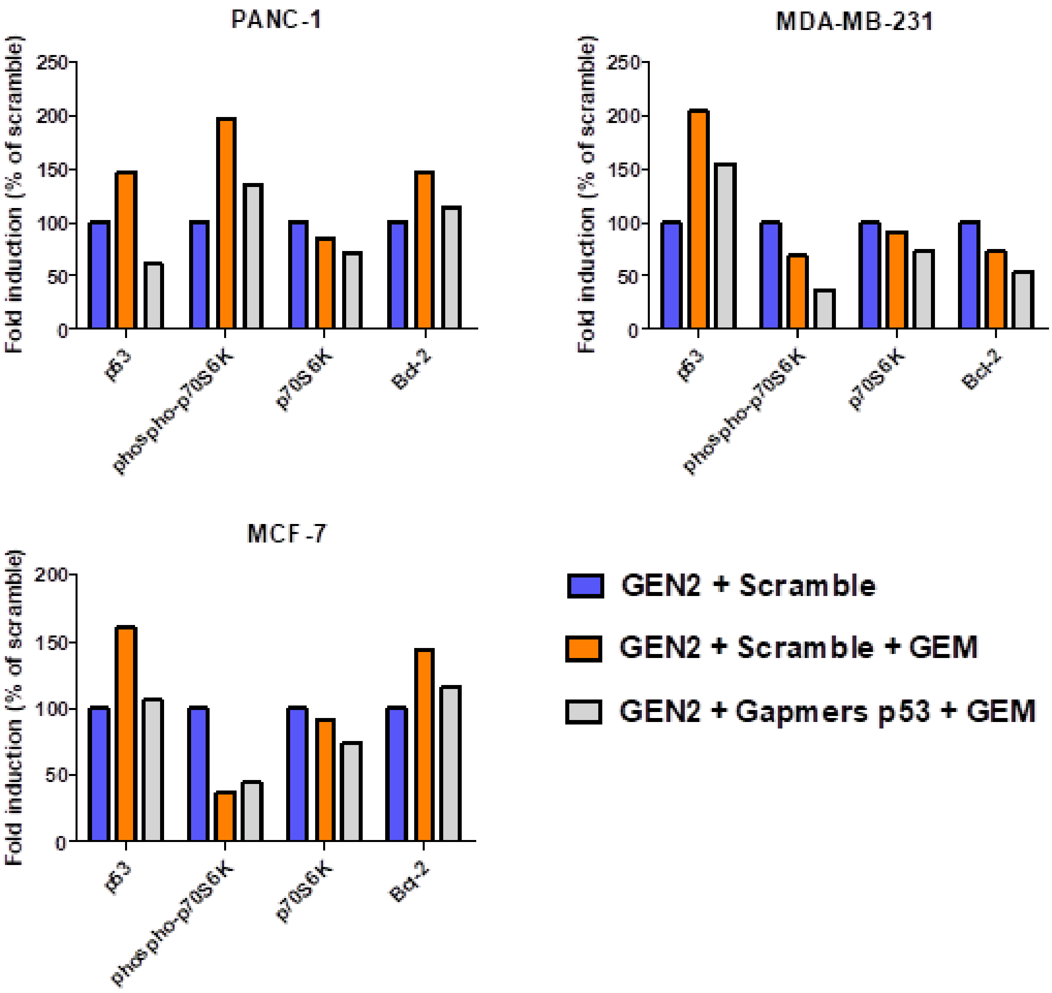

Figure S19. Quantitative analysis of p53/GAPDH, phospho(Ser371)-p70S6K/GAPDH, p70S6K/GAPDH and Bcl-2/GAPDH ratios. The bands shown in Figure 5D were scanned as digital peaks and the areas of the peaks were reported as fold change in percentage with respect to the scramble, as described in Material and Methods.

Instrumental analysis and Methods

General information

All the synthesized compounds have been analyzed by 1H and 13C using a Bruker DPX (400 MHz) spectrometer (Bruker, Mannheim, Germany). The quantification of the mass from the synthesized compounds have been performed at SIdI-UAM using electronic ionization (EI), electrospray ionization (ESI) and matrix-assisted laser ionization (MALDI). UV-Vis spectra were recorded in a quartz cuvette using a CARY 5000 spectrometer from Agilent (Santa Clara, CA, USA) at 25 °C. pH measurements were performed using a Mettler Toledo FiveEasy Plus pH-meter (Shanghai, China). The pH-meter was calibrated using 4.01, 7.01 and 10.01 pH standard buffers. An average value is obtained from three different measurements.

TEM

AuNPs were visualized using a 100 KeV JEM1010 (Jeol) at CBMSO-CSIC. Samples were prepared by placing a copper grid (AGAR.) over one drop of AuNPs for 2 min and drying the excess of the sample. The average diameter value was determined through an automated analysis of TEM images with Fiji ImageJ software (NHI, Bethesda, MD, USA).

Dynamic light scattering (DLS) and Zeta-Potential

DLS and Zeta potential measurements were performed using a Malvern Zetasizer Nano ZS. The scattered light was measured at an angle of 175° for DLS measurements and at 12.8° for zeta potential measurements using disposable folded capillary cells. The temperature of each sample was stabilized at 25 ± 0.1 °C for 45 s before the measurement. The measurements are performed using 1 mL of the gold nanoparticles without any dilution. For hydrodynamic size measurements, the polydispersity of the sample is took into account to discard samples with high heterogeneity. A normalized size and zeta potential distribution are obtained from three different measurements, and the mean value is indicated.

Gel retardation assay

The binding of PolyT(10)FAM to AuNPs GEN1 and GEN2 at different conditions was tested using the pellets and the supernatants obtained after the incubation stage. The samples (10 µL) were mixed with a gel loading buffer 6× (2 µL) at room temperature for 30 min. Then they were electrophoresed through a 3% (*w*/*v*) agarose gel in TAE (tris-acetate-EDTA) buffer at 90 mV and 500mA for 60 min. The gel was analyzed on a UV illuminator (search specifications).

Alamar Blue Viability Assay

A stock solution of resazurin sodium salt (Sigma-Aldrich, St. Louis, MO, USA) (1 mg/mL) in PBS was diluted 1% (*v*/*v*) in complete RPMI medium and added to the cells. After 3 h in the incubator (37 °C), the fluorescence was measured at 25 °C in a plate reader Synergy H4 Hybrid reader (BioTEK, Winooski, VT, USA), λ_{ex} = 550 nm, λ_{em} = 590 nm.The fluorescent intensity measurements were processed using the following Equation:% Cell viability = ((Sample data − Negative control)/(Positive control − Negative control)) × 100. The positive control corresponds with untreated cells. A resazurin solution without cells was used as negative control.

Western blot analysis

Cells were harvested, washed in PBS, and re-suspended in RIPA buffer (Tebu-BIO #AR0105) in the presence of a protease inhibitor cocktail (Thermo Scientific™ #A32955). After incubation on ice for 30 min, the lysates were centrifuged at 14000 × g for 10 min at 4 °C and the supernatant fractions were used for Western blot analysis. Protein concentration was measured by Bradford reagent (Bio-Rad protein assay) using bovine serum albumin as a standard. Protein extracts (30 µg/lane) were resolved on a 10% SDS-

polyacrylamide gel and electro-blotted onto PVDF membranes (Amersham™ Protran™ 0.45um NC). Membranes were blocked in 5% low-fat milk in TBST or 5% BSA (50 mM Tris pH 7.5, 0.9% NaCl, 0.1% Tween 20) for 1 h at room temperature and probed overnight at 4 °C with a rabbit polyclonal anti-Bcl-2 (1:1000) (Cell Signaling, #2872), rabbit polyclonal anti-p70 S6 Kinase (1:1000) (Cell Signaling, #9202), rabbit polyclonal anti-phospho- p70 S6 Kinase (Ser371) (1:1000) (Cell Signaling, #9208), mouse monoclonal anti-GAPDH (1:1000) (Santa Cruz, sc-47724) and mouse monoclonal anti-p53 (1:1000) (Santa Cruz, sc-47698). Horseradish peroxidase conjugated anti-mouse or anti-rabbit IgGs (1:5000 in blocking solution) (Santa Cruz, Spain) were used as secondary antibodies. Immunodetection was carried out using Bio-Rad chemiluminescent substrates and recorded using a ChemiDoc (Synergy, Cambridge, UK). ECL results were scanned and the amount of each protein band was quantified using NIH Image J software (NIH Image, Bethesda, MD, USA, http://rsb.info.nih.gov/nih-image/).

Fluorescence of polyT(10)FAM

Cells were plated in 96 or 12-well plates (5×10^3 or 8×10^4 cells/well, respectively) and 24 h later were treated with GEN2-AuNPs functionalized with 100 pmoles of polyT(10)FAM or transfected by Lipofectamine 2000 as described above. After 24 h from the treatment, cells were washed with 1X PBS (Phosphate Buffered Saline), pH 7.4 (VWR) and the fluorescence was analyzed. For microscopy images, the nucleus was stained with Hoechst 33342 and FAM fluorescence was measured using a Leica DMI3000 M inverted microscope (Leica, Wetzlar, Germany) at 20 × magnification. Images were analyzed using ImageJ software (NIH Image, Bethesda, MD, USA). FAM fluorescence was also measured by using a multimode plate reader (λ_{exc} = 395 nm and λ_{em} = 509 nm) (Synergy H4 Hybrid reader (BioTEK, Winooski, VT, USA).

Statistical analysis

Comparisons among groups were analyzed via independent-samples one-factor ANOVA test using GraphPad Prism 5.0 (GraphPad Software, Inc., San Diego, CA) Prism - GraphPad software,. All statistical data were obtained using a two-tailed student's t-test and homogeneity of variance tests (p values < 0.05 were considered significant).

Synthesis of linkers and polymer derivatives

2,5-dioxopyrrolidin-1-yl 5-(1,2-dithiolan-3-yl)pentanoate (Lipoic activated ester linker)[1]

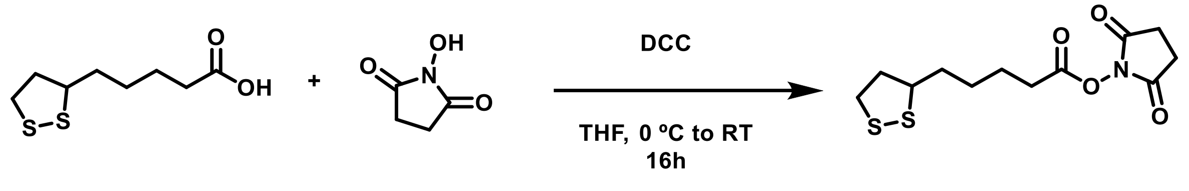

1 eq 1.2 eq 98%

To a solution of lipoic acid (3.6 g, 17.4 mmol) in tetrahydrofurane (THF, 65 mL) at 0 °C, a solution of N-hydroxysuccinimide (NHS) (2.2 g, 20.9 mmol) and dicyclohexylcarbodiimide (DCC) (4.32 g, 20.9 nmol) in THF (15 mL) was added under vigorous stirring. Then, the reaction was allowed to warm up to room temperature and the mixture was stirred for 16 h. Finally, the white precipitate was filtrated and the solvent removed under vacuum. The solid was dissolved in the minimum amount of ethyl acetate (AcOEt) and stored in the fridge for 6 h. The newly formed precipitate was removed as before. The solvent was removed again under vacuum, obtaining the desired lipoic ester (98% of yield, 5.17 g) derivative as a yellow oil. ^1H NMR ($CDCl_3$, 400 MHz): δ 3.54 (dq, 1H), 3.11 (m, 2H), 2.8 (s, 4H), 2.57 (t, 2H), 2.41 (m, 1H), 1.88 (m, 1H), 1.72 (m, 2H), 1.67 (m, 2H), 1.52

(m, 2H). ^{13}C NMR ($CDCl_3$, 101 MHz): δ 169.27, 168.44, 56.19, 40.25, 38.73, 34.78, 30.87, 28.32, 25.69, 24.45. MS (EI): *m/z* calculated for $C_{12}H_{17}NO_4S_2$ (M^+) 303.05, found 303.05.

(3-(pyridin-2-yldisulfanyl) propanoic acid) [2]

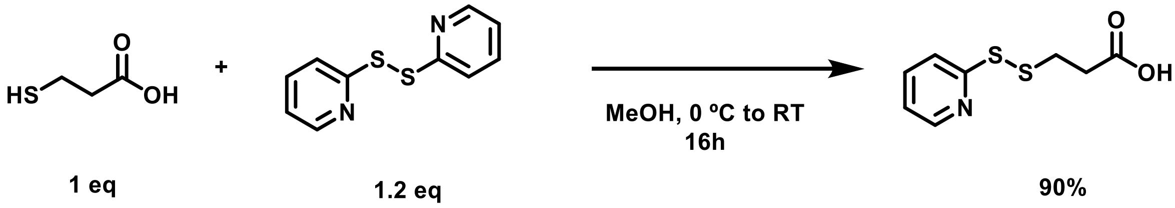

To a solution of mercaptopropionic acid (380.02 mg, 3.6 mmol) in methanol (MeOH, 30 mL) a mixture of 2-aldrithiol (1.213 g, 5.52 mmol) and acetic acid (0.150 mL) in MeOH (15 mL) was added and stirred for 16 h. Then, the solvent is removed under vacuum and the product was purified by column chromatography in silica gel with Hexane/AcOEt (1:1) affording a yellowish oil (90% of yield, 700.6 mg). ^{1}H NMR ($CDCl_3$, 400 MHz): δ 8.43 (d, 1H), 7.67 (m, 2H), 7.09 (td, 1H), 3.01 (t, 2H), 2.75 (t, 2H). ^{13}C NMR ($CDCl_3$, 101 MHz): δ 175.72, 159.52, 149.06, 137.69, 121.14, 120.29, 33.82, 33.55. MS (ESI): 216 (100) [M+H], 218 (5) [M+Na]. (ESI) calculated for C8H9NO2S2 (M+H) 216.01, found 216.014

2,5-dioxopyrrolidin-1-yl 3-(pyridin-2-yldisulfanyl)propanoate (SPDP linker) [3]

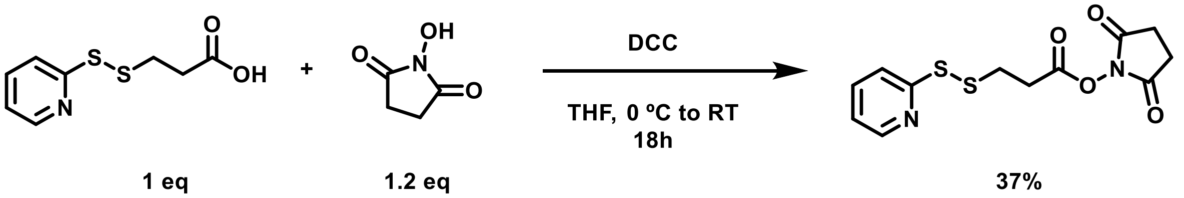

To a stirring solution (2-pyridyldithio)-propanoic acid (700.6 mg, 3.25 mmol) in THF (12 mL) at 0 °C, a solution of NHS (452 mg, 3.9 mmol) and DCC (805 mg, 3.9 nmol) in THF (3 mL) was added. The reaction was allowed to warm up to room temperature and stirred for 16 h. Then, the precipitated solid is filtrated and the solvent was removed under vacuum. The product was stored at -20°C for 6 h, then dissolved in dichloromethane (DCM) and the formed precipitate was filtrated. The solvent was removed again under vacuum obtaining a white solid corresponding to the ester (37% of yield, 375 mg). ^{1}H NMR ($CDCl_3$, 400 MHz): δ 8.37 (d, 1H), 7.58 (m, 2H), 7.01 (dt, 1H), 2.87 (m, 4H), 2.71 (s, 4H). ^{13}C NMR (CDCl3, 101 MHz): δ 169.05, 167.02, 159.15, 149.69, 137.54, 121.17, 120.11, 32.89, 30.95, 25.6. MS (ESI): 313 (100) [M+H], 335(47) [M+Na]. (ESI) calculated for C13H13NO4S2 (M+H) 312.03, found 313.01

Synthesis of LP-PEI (2000 MW) [4]

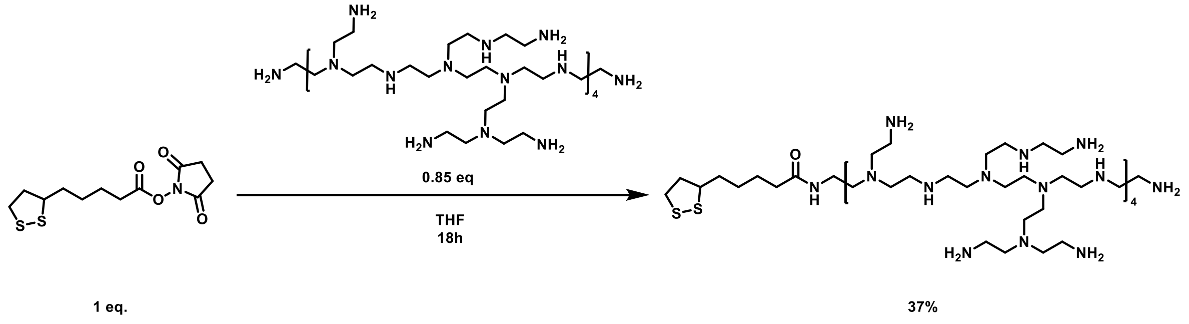

The procedure for the synthesis of LP-bPEI (2000 MW) is based on the synthesis of Zong *et al.* with some modifications. To a stirring solution of lipoic acid ester (9.37 mg, 0.0315 mmol) in THF (0.5 mL) at 0 °C a solution of polyethylenimine (2000 M.W.) (42 mg,

0.021 mmol) in THF was added. Once a grey oil-like compound appears, the solvent is removed under vacuum and re-dissolved in methanol (MeOH, 2 mL). The product was purified by dialysis using a 3.5 KDa. dialysis membrane during 16 h at 6 °C against distilled water. After this time, the solution turned cloudy and the solvent was removed under vacuum. The desired product was obtained as an orangish oil (37% of yield, 17 mg). ^{1}H NMR (DMSO-D6, 400 MHz): δ 8 (m, 3H), 4-2.82 (m, 199H), 1.88-0.98 (m, 57H). ^{13}C NMR(DMSO-D6, 121 MHz,) δ 173.02, 150.84, 149.86, 68.73, 60.85, 56.60, 52.29, 46.31, 35.76, 30.09, 29.62, 29.13, 28.41, 26.26, 25.63, 21.82, 15.19, 11.20. MS (MALDI): MS (MALDI): 130.1 corresponding to the polymer fragment $[C2H4NH]_3$.

Synthesis of PDP-PEI (2000 MW)

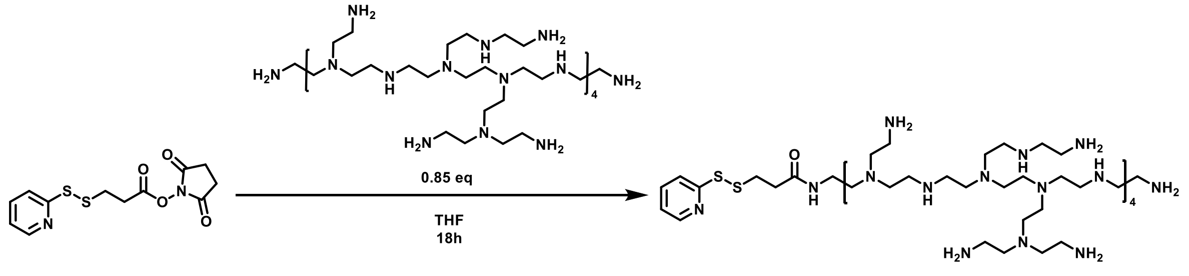

1 eq.

21%

To a solution of polyethylenimine (2000 M.W.) (37.5 mg, 0.018 mmol) in THF (1.5 mL) at 0 °C, a solution of SPDP (12 mg, 0.036 mmol) in THF (0.5 mL) was added. The reaction was allowed to warm up to room temperature under vigorous stirring for 16 h. Then, the solvent was removed under vacuum and re-dissolved in methanol (2 mL). The product was purified by dialysis using a 3.5 KDa. dialysis membrane during 16 h at 6 °C against distilled water. After this time, the solution turned cloudy and the solvent was removed under vacuum affording a greyish oil (21% of yield, 18.5 mg). ^{1}H NMR (DMSO-D6, 400 MHz): δ 8.49 (m, 2H), 8 (m, 37H), 7.77 (m, 4H), 7.30 (2H), 4.1-2.8 (m, 1436H), 2.02-0.84 (m, 414H). ^{13}C NMR (DMSO-D6, 101 MHz): δ 172.88, 164.18, 148.89, 137.25, 124.35, 120.73, 60.78, 53.50, 47.99, 33.64, 32.62, 29.47, 24.44, 21.73. MS (MALDI): 169.1 and 212.2, corresponding to polymer fragments due to their difference of 43 units, which corresponds with the monomer [C2H4NH].

Synthesis of LP-PEG (3000 MW) [5]

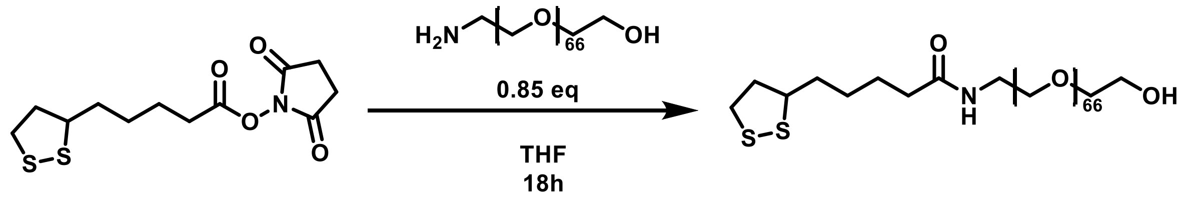

1 eq

45%

The procedure for the synthesis of LP-PEG (3000 MW) is based on the synthesis of Pyo *et al.* with some modifications. To a solution of lipoic acid ester (5.7 mg, 0.087mmol) in THF (0.5 mL) at room temperature a solution of polyethylenglycol (50 mg, 0.016) THF (0.5 mL) was added and stirred for 16 h. Then, the solvent was removed under vacuum and re-dissolved in methanol (MeOH, 2 mL). The product was purified by dialysis using a 3.5 KDa. dialysis membrane for 16 h at 6 °C against distilled water. Then, the solvent was removed under vacuum, obtaining a yellowish solid as the desired compound. (45% of yield, 24 mg). ^{1}H NMR ($CDCl_3$, 400 MHz): δ 3.74 (s, 258H), 3.42 (t, 2H), 3.24 (dd.47 (m, 1H), 2.53 (t, 2H), 2,3(t,2H), 2.03 (m, 1H), 1.79 (m, 1H), 1.66 (m, 4H), 1.46 (m, 2H). ^{13}C NMR ($CDCl_3$, 400 MHz): δ 173.1, 171.9, 70.6, 56.4, 40.3, 39.28, 38.5, 36.3, 34.7, 33.51, 29.8, 25.5, 24.8. MS (MALDI): MS (MALDI): *m/z* calculated for $C_8H_{13}OS_2(PEG)_{66}$ 3047.7, found 3046.8

References

1. Porcaro, F.; Miao, Y.; Kota, R.; Haun, J.B.; Polzonetti, G.; Battocchio, C.; Gratton, E. Fluctuation Spectroscopy Analysis of Glucose Capped Gold Nanoparticles. *Langmuir* **2016**, *32*, 13409–13417. https://doi.org/10.1021/acs.langmuir.6b02545.
2. Biscans, A.; Rouanet, S.; Vasseur, J.J.; Dupouy, C.; Debart, F. A Versatile Post-Synthetic Method on a Solid Support for the Synthesis of RNA Containing Reduction-Responsive Modifications. *Org. Biomol. Chem.* **2016**, *14*, 7010–7017. https://doi.org/10.1039/c6ob01272h.
3. Liu, Y.; Zhou, Z.; Lin, X.; Xiong, X.; Zhou, R.; Zhou, M.; Huang, Y. Enhanced Reactive Oxygen Species Generation by Mitochondria Targeting of Anticancer Drug to Overcome Tumor Multidrug Resistance. *Biomacromolecules* **2019**, *20*, 3755–3766. https://doi.org/10.1021/acs.biomac.9b00800.
4. Zheng, M.; Zhong, Y.; Meng, F.; Peng, R.; Zhong, Z. Lipoic Acid Modified Low Molecular Weight Polyethylenimine Mediates Non-toxic and Highly Potent in Vitro Gene Transfection. *Mol. Pharm.* **2011**, *8*, 2434–2443. https://doi.org/10.1021/mp2003797.
5. Kim, J.-H.; Sim, G.-S.; Bae, J.-T.; Oh, J.-Y.; Lee, G.-S.; Lee, D.-H.; Lee, B.-C.; Pyo, H.-B. Synthesis and Anti-Melanogenic Effects of Lipoic Acid-Polyethylene Glycol Ester. *J. Pharm. Pharmacol.* **2008**, *60*, 863–870. https://doi.org/10.1211/jpp.60.7.0007.
